# Lysergic Acid Diethylamide Induces Transcriptional Changes Opposing Signatures of Brain Ageing and Neurodegeneration

**DOI:** 10.1111/acel.70793

**Published:** 2026-11-10

**Authors:** Aurora Savino, Carla Liaci, Ilaria Bertani, Simona Rando, Mattia Camera, Giorgio R. Merlo, Lidia Avalle, Valeria Poli, Nereo Kalebic, Francesco Iorio

**Affiliations:** ^1^ Human Technopole Milan Italy; ^2^ Department of Molecular Biotechnology and Health Sciences, Molecular Biotechnology Center University of Turin Turin Italy; ^3^ Department of Science and Technological Innovation University of Piemonte Orientale Alessandria Italy

**Keywords:** amyloid beta‐peptides, brain ageing, gene expression, lysergic acid diethylamide, neurodegeneration, transcriptome

## Abstract

Psychedelic compounds such as lysergic acid diethylamide (LSD) are increasingly studied for their neuroplastic effects and potential relevance to brain ageing and neurodegenerative conditions, including dementia. These processes are characterised by transcriptional changes involving progressive synaptic dysfunction and altered neuron–glia interactions. In computational drug discovery, the transcriptional signature reversion principle proposes that compounds inducing gene expression states opposite to disease‐associated programmes may have therapeutic potential by counteracting disease‐associated phenotypes. We therefore asked whether LSD‐induced transcriptional changes oppose gene expression programmes associated with brain ageing and dementia, thereby providing a data‐driven rationale for its potential relevance to age‐associated cognitive decline and a starting point for investigating the underlying molecular mechanisms. Combining cross‐species transcriptomic analyses with experimental validation, we show that transcriptional profiles induced by chronic LSD treatment in rats are strongly anti‐correlated with ageing‐associated gene expression changes in the human prefrontal cortex and, to a lesser extent, with dementia‐associated programmes. This relationship is reproducible across datasets and species and is significantly stronger compared to a broad panel of pharmacological perturbations. Independent acute LSD treatment in mice recapitulated the opposition to ageing‐associated transcriptional signatures across multiple doses. Moreover, short‐term LSD treatment attenuated amyloid‐β‐induced structural, molecular and transcriptional alterations in primary cortical cultures. Together, these findings provide a transcriptionally grounded rationale for investigating LSD and related compounds in brain ageing and neurodegeneration, while identifying molecular and cellular programmes that can guide future mechanistic and therapeutic studies.

## Graphical abstract

Lysergic acid diethylamide (LSD) induces transcriptional programmes in the rodent prefrontal cortex that oppose signatures of human brain ageing and neurodegeneration. In primary cortical cultures, LSD also attenuates amyloid‐β‐induced alterations. Consistent with the signature reversion principle, these findings provide a data‐driven rationale for investigating psychedelic‐associated pathways in neurodegenerative contexts.

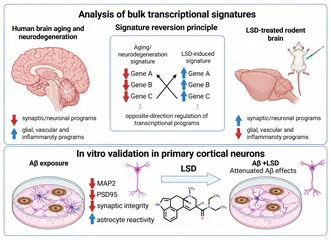

## 1 Introduction

Psychedelic compounds are increasingly recognised as promising therapeutic agents for a range of psychiatric disorders (De Gregorio et al. 2021). Among the most extensively studied are psilocybin, lysergic acid diethylamide (LSD), 2,5‐dimethoxy‐4‐iodoamphetamine (DOI) and dimethyltryptamine (DMT). Despite their chemical diversity, these substances share a common pharmacological feature: high‐affinity binding to and activation of the serotonin 5‐HT2A receptor, which is widely considered central to their psychoactive and therapeutic effects (Banks et al. 2021). A growing body of evidence indicates that classic psychedelics function as psychoplastogens, rapidly inducing both structural and functional neuroplasticity (Inserra et al. 2021; Banks et al. 2021; Ly et al. 2018; Calder and Hasler 2023; Aleksandrova and Phillips 2021). Consistently, these compounds promote neurogenesis (Calleja‐Conde et al. 2026) both in vitro (Morales‐García et al. 2017) and in vivo (Lima da Cruz et al. 2018), drive dendritic spine remodelling in pyramidal neurons (Ly et al. 2018; Yoshida et al. 2011; Shao et al. 2021; Jones et al. 2009) and upregulate neurotrophic factors such as brain‐derived neurotrophic factor (BDNF; de Vos et al. 2021).

These convergent cellular and molecular effects suggest that psychedelics engage processes involved in synaptic maintenance and neuronal resilience, both of which are progressively compromised during brain ageing and more severely disrupted in neurodegenerative disorders such as Alzheimer's disease. On this basis, psychedelics have been proposed as potential therapeutic agents to slow cognitive decline and mitigate the risk or progression of Alzheimer's disease (Saeger and Olson 2022; Aday et al. 2020; Winkelman et al. 2023; Haniff et al. 2024; Kozlowska et al. 2022; Sinha et al. 2024; Androni et al. 2025). However, clinical investigation in this area remains at an early stage, with completed studies limited to Phase I trials assessing safety and tolerability (Family et al. 2020; Bouchet et al. 2024), and an ongoing pilot trial evaluating the potential of psilocybin for the treatment of early‐stage dementia (ClinicalTrials.gov identifier: NCT04123314).

More direct links between psychedelics and ageing biology have recently emerged. Psilocin, the active metabolite of psilocybin, has been shown to extend cellular lifespan in vitro without inducing malignant transformation, consistent with a delay of, rather than an escape from, replicative senescence (Kato et al. 2025). In the same study, intermittent administration of psilocybin to aged mice significantly improved survival, suggesting that psychedelic compounds may also influence biological processes associated with ageing in vivo (Kato et al. 2025). Together, these findings raise the possibility that psychedelics modulate conserved molecular programmes associated with ageing.

Foundational work in computational drug discovery and repositioning has established the principle of *transcriptional signature reversion*, whereby compounds inducing gene expression states opposite to disease‐associated signatures may have therapeutic potential (Arloth et al. 2015; Iorio et al. 2013; Lamb et al. 2006; Dudley et al. 2011; Kunkel et al. 2011; Dönertaş et al. 2018; Oskotsky et al. 2026; Pushpakom et al. 2018). Importantly, this relationship is not restricted to molecular similarity: experimental studies have shown that pharmacological reversal of disease‐associated transcriptional states can be accompanied by improvement of disease‐relevant physiological phenotypes, as demonstrated in a mouse model of dyslipidemia (Wagner et al. 2015).

Building on these premises, we asked whether LSD induces gene expression changes opposite to those associated with brain ageing and dementia, and whether such a relationship could provide a data‐driven rationale for its potential relevance to age‐associated cognitive decline. We first compared transcriptional signatures elicited by chronic LSD treatment in the rat medial prefrontal cortex with age‐ and dementia‐associated gene expression changes in the human prefrontal cortex, leveraging large‐scale transcriptomic datasets and public repositories. To assess whether this relationship was reproducible across species and treatment regimens, we then generated an independent transcriptomic dataset from the mouse prefrontal cortex following acute LSD administration at multiple doses.

Given the experimental precedent linking transcriptional signature reversion to phenotypic modulation, and because most dementia datasets included in our analysis were derived from Alzheimer's disease patients, we next asked whether the transcriptional opposition observed for LSD extended to AD‐relevant cellular alterations. We therefore treated primary mouse cortical cultures exposed to amyloid‐β with LSD and assessed dendritic architecture, synaptic and cytoskeletal markers, astrocyte activation and transcriptional responses.

## 2 Results

### 2.1 LSD‐Induced Transcriptional Changes Oppose Age‐Associated Gene Expression Programmes in the Human Prefrontal Cortex

We analysed the largest RNA‐seq dataset to date of chronic LSD‐induced gene expression changes in the rat medial prefrontal cortex (mPFC) (Martin et al. 2014; Savino and Nichols 2022), with the goal of determining whether the transcriptional signatures induced by LSD could be classified as opposing ageing‐associated gene expression programmes in the human brain.

To derive a consensus transcriptomic signature of human brain ageing, we queried the Gene Expression Omnibus (GEO) database (Barrett et al. 2013) for prefrontal cortex (PFC) gene expression datasets with annotated donor age (Tables S1–S3), retrieving gene expression data from healthy individuals, encompassing 3086 samples spanning ages from 2 days to 106 years (Figure S1).

We structured the initial analysis in three steps. First, we derived a genome‐wide ranked list of genes from an integrated human dorsolateral prefrontal cortex (DLPFC) dataset by ordering genes according to the correlation between their expression levels and donor age, from those most positively correlated with age to those most negatively correlated. This ranked list therefore captured a continuum of age‐associated transcriptional change across the genome and was used to test whether chronic LSD‐induced transcriptional signatures were directionally opposed to age‐related gene expression patterns. Second, to assess robustness across heterogeneous datasets, we repeated the same GSEA‐based analysis within each ageing dataset separately, using dataset‐specific age‐expression correlations. Third, we compared the resulting ageing‐associated patterns with an independent published brain ageing signature to confirm that the curated datasets captured canonical age‐related transcriptional changes.

We started our transcriptomic data integration by removing latent variables representing confounders not linked to our variable of interest (age). To maximise comparability, we restricted the analysis to the dorsolateral prefrontal cortex (DLPFC), which provided the largest number of samples (*n* = 681; Figures S2–S4). Across studies, we retained 4893 genes consistently measured in all datasets.

We then computed gene‐wise Pearson correlations between expression levels and chronological age across the integrated dataset and ranked all genes according to their correlation with age. This ranked list, therefore, defined a genome‐wide ageing trajectory, ranging from genes upregulated to genes downregulated with age.

To test whether LSD‐induced transcriptional changes oppose ageing‐associated programmes, we performed gene set enrichment analysis (GSEA) (Subramanian et al. 2005), using the age‐correlation‐ranked list as background gene‐list and the sets of genes up‐ or downregulated by chronic LSD treatment in rats (Martin et al. 2014) as query signatures (Section 4). This allowed us to test whether genes upregulated by LSD were preferentially enriched among genes whose expression decreases with age, and conversely whether LSD‐downregulated genes were enriched among genes whose expression increases with age, consistent with the transcriptional signature reversal principle established in computational drug discovery.

We observed that genes upregulated by LSD were significantly enriched among those whose expression was negatively correlated with age, located towards the lower end of the age‐ranked gene list (GSEA *p* = 1.8 × 10^−25^, normalised enrichment score (NES) = −5.81, Figure 1A). Consistently, genes downregulated by LSD were enriched among those positively correlated with age, located towards the upper end of the ranked list (GSEA *p* = 3.6 × 10^−44^, NES = 5.54, Figure 1A).

**FIGURE 1:**
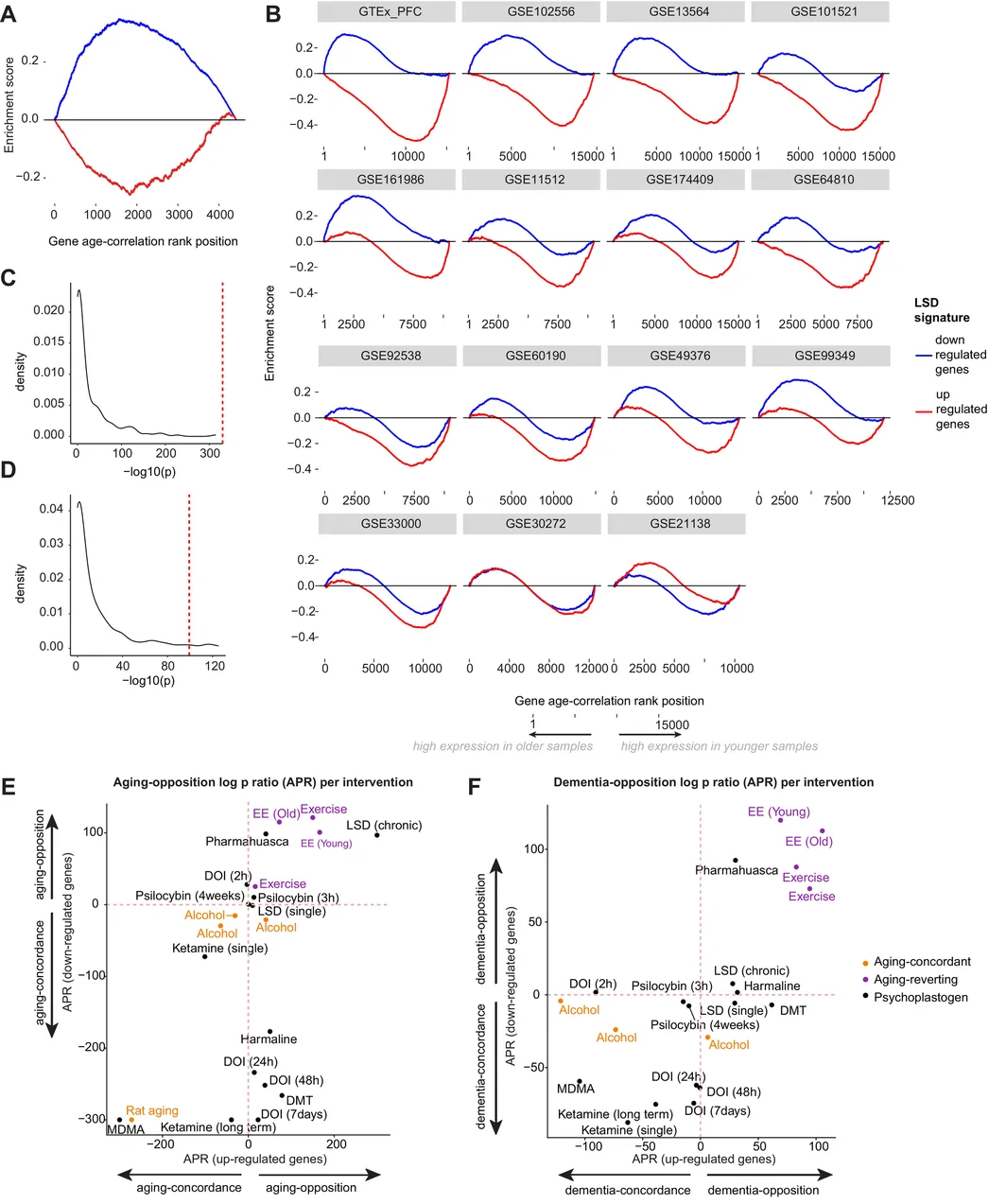
(A) Gene Set Enrichment Analysis (GSEA) of genes ranked according to their correlation with age in the integrated dorsolateral prefrontal cortex (DLPFC) datasets using differentially expressed genes (DEGs) upon chronic LSD administration (up‐ or down‐regulated, as indicated by the different colours) as query signatures. Up‐regulated genes *p*‐value = 1.8 × 10^−25^, normalised enrichment score (NES) = −5.81; down‐regulated genes *p*‐value = 3.6 × 10^−44^, NES = 5.54. (B) Gene Set Enrichment Analysis (GSEA) of genes ranked according to the correlation of their expression with age across prefrontal cortex datasets using differentially expressed genes upon chronic LSD administration (up‐ or down‐regulated, as indicated by the different colours) as query signatures. (C, D) Distribution of age‐signature opposition *p*‐values for drugs profiled in the Connectivity Map and tested in neural progenitor cells, computed using sets of drug‐dependent upregulated (C) or downregulated (D) genes as query signatures, following the same GSEA‐based framework summarised in panel (A). The red dashed line indicates the *p*‐value obtained for chronic LSD exposure. (E) Summary of transcriptional age‐signature opposition and concordance properties of various treatments: A set of psychoplastogens in black, ageing‐reverting comparators in purple, ageing‐concordant comparators in orange. EE = enriched environment. For each intervention dataset (psychoplastogen, exercise, enriched environment), we computed the meta‐analytic *p*‐value for ageing opposition and concordance across human ageing datasets, separately for genes up‐ and down‐regulated upon the intervention. We then plotted the log‐ratio between opposition and concordance *p*‐values. Positive values indicate stronger evidence for ageing‐opposition than ageing‐concordance, whereas negative values indicate stronger evidence for ageing‐concordance. (F) Same plot as E, using dementia datasets instead of ageing datasets.

To determine whether this pattern was reproducible across individual studies rather than being driven by the integrated analysis or by the procedures required to harmonise heterogeneous datasets, we next repeated the GSEA separately within each ageing dataset. For each study, we independently ranked genes according to their correlation with age and tested the LSD‐regulated gene sets against that dataset‐specific ranking. This approach allowed us to assess the consistency of the transcriptional opposition across cohorts and platforms while avoiding dependence on cross‐study batch correction. It also increased gene coverage by removing the requirement that genes be measured across all datasets and enabled inclusion of medial prefrontal cortex (mPFC) datasets, matching the brain region profiled in the chronic LSD study.

Despite substantial inter‐dataset heterogeneity, most datasets exhibited a consistent overall enrichment pattern of the LSD induced signature (Figure 1B; Figure S5; Table S4), with a slightly stronger and more reproducible opposition signal for LSD upregulated rather than downregulated genes.

We combined dataset‐specific GSEA *p*‐values using Fisher's method (Fisher 1992), which aggregates evidence across independent tests without requiring data integration or normalisation across sources. This approach is robust to inter‐dataset heterogeneity in platform, preprocessing and sample composition, as each dataset contributes an independent statistical observation rather than individual samples to a pooled analysis. This yielded a combined *p*‐value < 2.2 × 10^−308^ for upregulated genes and 5.2 × 10^−100^ for downregulated genes. The median dataset‐specific NES values were −1.96 for upregulated and 1.22 for downregulated gene sets, indicating consistent effect sizes across datasets (Figure 1B; Figure S5; Table S4). We note that Fisher's combined *p*‐value reflects the consistency of the signal across datasets rather than a single‐comparison statistical test; individual dataset‐level results are reported in Figure 1B to allow assessment of magnitude, direction and consistency across studies.

These results further support the conclusion that LSD‐induced transcriptional changes are directionally opposed to age‐associated transcriptional signatures.

To assess the robustness of our findings, we evaluated whether the age‐associated transcriptional patterns captured by the datasets analysed here were consistent with an independent, previously published brain ageing signature (Dönertaş et al. 2018).

Specifically, we repeated a GSEA using genes reported to increase or decrease expression with ageing in that study as query signatures. We observed a strong concordance across datasets, as reflected by highly significant Fisher‐aggregated *p*‐values (3.2 × 10^−227^ and 7.8 × 10^−322^ for genes with increasing/decreasing expression with age, respectively; Figure S6), indicating that our datasets robustly capture canonical ageing‐related transcriptional effects.

Together, these analyses show that chronic LSD‐induced transcriptional changes consistently oppose age‐associated gene expression patterns across diverse ageing datasets, and that these patterns are concordant with an independently derived brain ageing signature.

### 2.2 LSD Shows the Strongest Age‐Associated Transcriptional Anti‐Correlation Among Psychoplastogens and Other Pharmacological Perturbations

To determine whether the inverse association between LSD and ageing signatures was specific rather than a general feature of transcriptional perturbations, we compared LSD with a broad panel of pharmacological interventions. Specifically, we analysed transcriptional signatures from 193 compounds profiled in neural progenitor cells within the Connectivity Map (CMap) resource (Subramanian et al. 2017), applying the same GSEA‐based framework used for LSD. Under a non‐specific or confounded scenario, a substantial fraction of drug‐induced signatures would be expected to exhibit comparable ageing‐signature opposition signal. Instead, we found that LSD ranked above all tested compounds when considering upregulated genes, and above 97% of compounds when considering downregulated genes, based on Fisher‐aggregated *p*‐values (Figure 1C,D; Figures S7 and S8; Section 4), supporting a high degree of specificity of the LSD‐associated transcriptional anti‐correlation with ageing signatures.

We next asked whether the anti‐correlation with ageing‐signatures was specific to LSD or could also be observed for other psychedelics, entactogens and dissociative compounds (collectively referred to as ‘psychoplastogens’; Table S5) for which rodent prefrontal cortex transcriptomic data were available. As benchmarks, we selected physical exercise and enriched environment (EE), expected to produce ageing‐opposing transcriptional profiles, and chronic ethanol exposure, expected to produce ageing‐concordant profiles, based on evidence that chronic alcohol exposure promotes transcriptional changes convergent with brain ageing, including neuroinflammatory gene induction and synaptic gene downregulation in the prefrontal cortex (Sun et al. 2023; Joshi et al. 2024). In addition, we include Rat ageing as reference (Table S6).

For each condition, we identified differentially expressed genes (FDR < 0.05) and evaluated their directional enrichment along ageing‐associated ranked gene lists using the same GSEA‐based framework applied to LSD (Section 4). Dataset‐specific statistics were aggregated using Fisher's method, and we quantified the net directionality of each intervention as the ageing‐signature opposition *p*‐value ratio (APR), reflecting the relative strength of ageing‐opposing versus ageing‐concordant signals (Figure 1E; Section 4).

Chronic LSD exposure exhibited the strongest overall ageing‐opposition (average APR across up‐ and down‐regulated genes = 198), exceeding that observed for physical exercise and EE, which, as expected, also showed positive APR values (largest average APR = 136; Figure 1E; Tables S4 and S7). In contrast, the rat ageing signature displayed the most pronounced negative average APR (−286), consistent with strong ageing‐concordant activity. Ethanol treatment similarly produced an ageing‐concordant transcriptional profile (largest average APR = −47).

Chronic LSD did not show a greater average transcriptional effect than the other interventions. Mean logFCs among LSD‐regulated genes were +0.29 and −0.27 for up‐ and down‐regulated genes, respectively, corresponding to an average absolute logFC of 0.28 (Table S8). Notably, the ageing‐reverting comparators exercise and enriched environment also showed relatively modest transcriptional changes (mean absolute logFC across comparator datasets = 0.33), despite their expected positive ageing‐opposition profiles, while ageing‐concordant comparators showed a similar average magnitude (0.27).

We therefore also quantified the cumulative transcriptional opposition (CTO), which incorporates both the number of directionally opposing or concordant DEGs and the magnitude of their intervention‐induced logFCs. Chronic LSD showed a strong positive CTO for both up‐ and down‐regulated genes (CTO = 70 and 39, respectively; total CTO = 109, Figure S9A; Tables S9 and 10).

Among the remaining psychoplastogens, only repeated pharmahuasca exposure (average APR = 70) and, to a much lesser extent, acute psilocybin treatment assessed 3 h post‐administration (average APR = 12) showed mild ageing‐opposition signal compared to that of the controls. Most other interventions exhibited ageing‐concordant profiles, with MDMA displaying the strongest effect (average APR < −300; Figure 1E).

Because neurodegenerative diseases display several age‐associated transcriptional programmes, we next asked whether LSD‐induced signatures were directionally opposed to dementia‐associated gene expression changes. We extended the analysis to dementia‐associated signatures, mostly from Alzheimer's Disease patients but also including dementia with Lewy bodies, Parkinson's disease dementia and Huntington's disease (Table S11). Since the dementia datasets compare diagnostic groups rather than continuous age trajectories, we summarised treatment‐associated gene set activity in control and dementia samples using single‐sample GSEA, and summarised group differences through Cohen's d before aggregating evidence across datasets (Figure S10; Section 4).

Chronic LSD showed an overall dementia‐opposition profile (average APR = 18), with a stronger signal for LSD‐upregulated genes (APR = 28) than for LSD‐downregulated genes (APR = 8; Figure 1F; Figure S9; Tables S12 and S13). This directional relationship was also evident when considering the magnitude of transcriptional changes: chronic LSD showed positive cumulative transcriptional opposition for both up‐ and down‐regulated genes (CTO = 41 and 26, respectively; total CTO = 67; Figure S9B; Tables S9 and 14).

Repeated pharmahuasca also showed a marked dementia‐opposition profile, with a stronger signal for its downregulated gene signature (APR = 92) than for its upregulated signature (APR = 30; Figure 1F; Figure S10; Tables S12 and S13).

Finally, we performed two external validation analyses. First, using two independent ageing datasets not included in the GEO query, we confirmed that genes regulated by chronic LSD treatment showed expression changes opposite to age‐associated transcriptional changes (Figure S11A; Section 4). Second, using the Mount Sinai Brain Bank cohort, we observed that chronic LSD‐regulated genes displayed expression changes opposite to those associated with increasing Alzheimer's disease severity (Figure S11B). These analyses further support the transcriptional opposition between LSD‐regulated genes and ageing‐ or neurodegeneration‐associated signatures.

### 2.3 Acute LSD Treatment in Mice Reproduces Transcriptional Patterns Anti‐Correlated With Ageing Signatures Across Doses

Next, we generated an independent transcriptomic dataset from mice following a single intraperitoneal administration of LSD (Figure 2A). We tested multiple doses (50, 100, 200 and 500 μg kg^−1^) and profiled gene expression in the PFC 90 min after treatment. Across all doses, DEGs (FDR < 0.05) exhibited transcriptional changes opposing age‐associated signatures, for both up‐ and downregulated gene sets (average APR across all concentrations = 100, Figure 2B, Tables S15 and S16). Notably, higher doses did not produce stronger ageing‐signature opposition, with the most robust signal observed at the intermediate dose of 200 μg kg^−1^ (average APR = 265). This pattern is consistent with the anti‐correlation observed following chronic LSD treatment in rats, suggesting that the transcriptional opposition to ageing signatures may emerge across treatment regimens.

**FIGURE 2:**
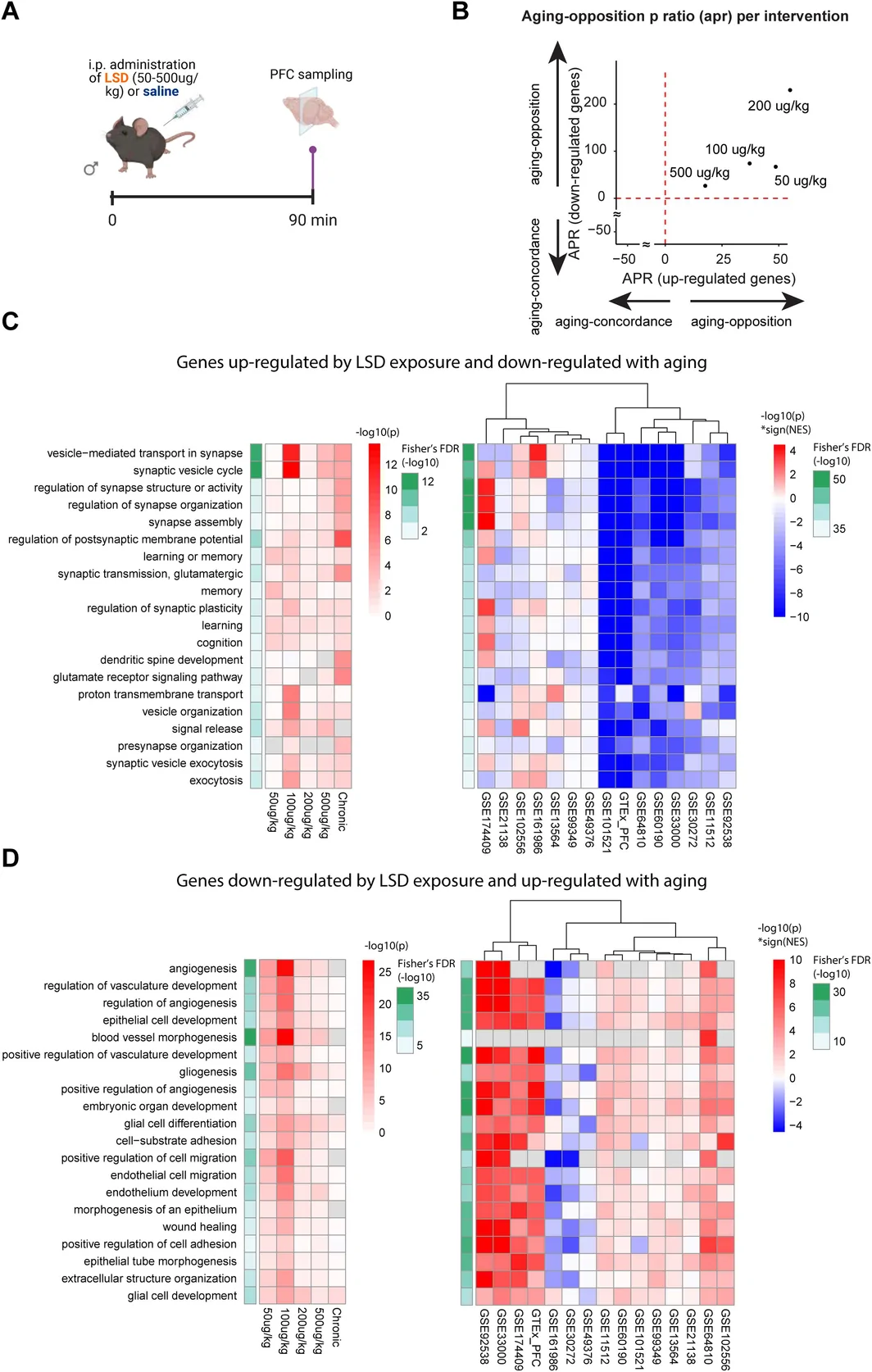
(A) Schematic of in vivo treatment and sampling. (B) Summary of transcriptional ageing‐signature opposition and concordance of acute LSD treatment at different dosages in mice. Each point corresponds to the effect in mouse prefrontal cortex of a different dose of LSD, with coordinates on the *x* and *y* axis indicating, respectively, the APR up‐regulated and down‐regulated genes (Methods), as in Figure 1E. (C, D) Top 20 Gene Ontology (GO) categories significantly enriched among genes exhibiting inverse regulation between LSD treatment and ageing: Genes upregulated by LSD and negatively correlated with age (C), and genes downregulated by LSD and positively correlated with age (D). Left: The colour in the heatmap indicates the GSEA *p*‐value (−log10) with the sign determined by the normalised enrichment score (NES). Red, highly significant positive enrichments; blue, highly significant negative enrichments. The green bar on the left indicates the overall FDR (−log10) aggregated across datasets with the Fisher's method. Right: GO enrichment *p*‐value (−log10) for genes up‐ (C) and down‐regulated (D) by LSD upon different treatment regimens. The green bar on the left indicates the overall *p*‐value (−log10) combining the *p*‐values obtained for each regimen. i.p. = intraperitoneal.

### 2.4 LSD‐Associated Ageing‐Signature Opposition Involves Synaptic and Dendritic Transcriptional Programmes

To elucidate the biological processes underlying LSD‐associated age‐signature anti‐correlation detected across both chronic and acute treatment in rats and mice, respectively, we performed a Gene Ontology (GO) enrichment analysis on genes showing inverse regulation between LSD treatment and ageing (i.e., genes upregulated by LSD and negatively correlated with age, and vice versa). Consistent with prior studies of brain ageing (Ham and Lee 2020), GO‐categories related to synapse‐ and dendrite‐related processes—typically downregulated with ageing—were enriched among genes upregulated by LSD (Figure 2C), supporting the biological coherence of the observed anti‐correlation (Table S17). Conversely, categories associated with angiogenesis and gliogenesis, which are known to increase with ageing, were preferentially downregulated following LSD treatment (Figure 2D; Table S18). Comparable patterns were observed when repeating the analysis using dementia‐associated transcriptional signatures instead of ageing datasets (Figure S12; Tables S19 and S20), indicating that the transcriptional changes associated with LSD are opposite to the molecular pathways implicated in both physiological brain ageing and neurodegenerative progression.

### 2.5 LSD Attenuates Amyloid‐β‐Induced Structural and Molecular Deficits in Primary Cortical Neurons

Converging evidence from Alzheimer's disease (AD) models indicates that synaptic and cytoskeletal alterations precede overt neuronal loss and contribute to cognitive decline. Amyloid‐β (Aβ) disrupts dendritic architecture, arborization and synaptic plasticity, including downregulation of Postsynaptic Density Protein 95 (PSD95), while also perturbing neuronal microtubule organisation and axonal transport (Lopes et al. 2022; Staurenghi et al. 2021). In parallel, Aβ promotes maladaptive astrocyte activation, characterised by increased Glial Fibrillary Acidic Protein (GFAP) expression and synaptotoxic signalling (Davis et al. 2021). Together, Aβ‐induced dendritic simplification, loss of synaptic integrity, cytoskeletal disruption and reactive astrocytosis represent well‐established cellular features of AD (Davis et al. 2021; Lopes et al. 2022).

Because pharmacological reversal of disease‐associated transcriptional states has previously been shown to coincide with improvement of disease‐relevant phenotypes (Wagner et al. 2015), and because most dementia datasets included in our analysis were derived from Alzheimer's disease patients, we next asked whether the transcriptional opposition observed for LSD was accompanied by modulation of AD‐relevant cellular alterations. To address this, we examined the effects of LSD in a well‐established in vitro model of Aβ‐induced neurodegeneration (Beretta et al. 2020). We cultured primary cortical neurons together with residual astrocytes derived from postnatal Day 2 (P2) mice for 20 days and exposed them to 100 nM Aβ for 3 h, followed by LSD or vehicle treatment for an additional 24 h. As a proxy for neuronal morphology and arborization, we quantified by immunofluorescence the area positive for microtubule‐associated protein 2 (MAP2).

Aβ exposure markedly reduced MAP2‐positive dendritic architecture, whereas subsequent LSD treatment significantly restored it towards control levels (Figure 3A,B). LSD treatment alone did not alter neuronal architecture, supporting a selective attenuation of the Aβ‐induced structural phenotype.

**FIGURE 3:**
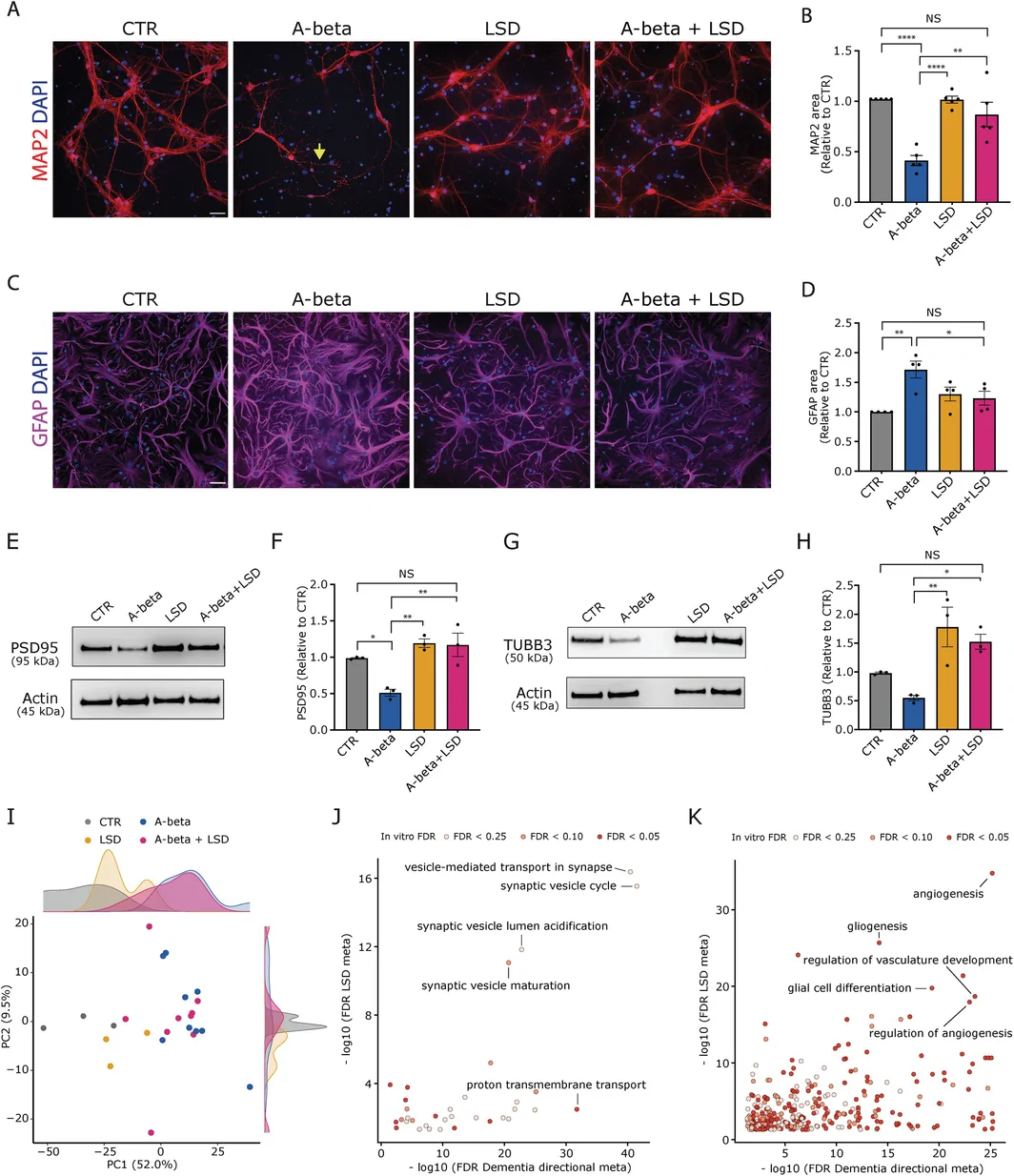
LSD attenuates Aβ‐induced alterations in primary mouse neurons and astrocytes. P2 mouse cortical neurons were grown in culture for 20 days, after which they were treated with 100 nM Aβ for 3 h, followed by 10 μM LSD or vehicle treatment for a further 24 h, generating four different conditions: CTR (no Aβ, DMSO); LSD; Aβ; and Aβ + LSD. (A) Immunofluorescence (IF) images for MAP2 (red) along with DAPI staining of treated cortical neurons. Scale bar, 50 μm. The arrow in Aβ indicates fragmented neuronal processes. (B) Quantification of the area of the MAP2 IF signal normalised to the control, average of five independent experiments. Barplots show means ± Standard Error Mean (SEM) of *N* = 5 independent experiments with 4–6 areas of interest imaged per sample; Statistical analyses were performed for indicated comparisons by Ordinary one‐way ANOVA (**p < 0.01; *p < 0.05). (C) IF images for GFAP (magenta) along with DAPI staining of treated astrocytes present in the cortical neuron culture. Scale bar, 50 μm. (D) Quantification of the area of the GFAP IF signal normalised to the control, average of four independent experiments, as a measure of astrocyte activation. Barplots show means ± SEM of *N* = 4 independent experiments; Statistical analyses were performed for all comparisons by Ordinary one‐way ANOVA; only statistically significant differences are indicated (****p < 0.0001; **p < 0.01). (E) Immunoblot for PSD95. Actin was used as a loading control. (F) Quantification of the densitometric analysis of PSD95 band normalised to actin band signal and expressed relative to the control average. Barplots show means ± SEM of *N* = 3 independent experiments; Statistical analyses were performed for all comparisons by Ordinary one‐way ANOVA; only statistically significant differences are indicated (**p < 0.01; *p < 0.05). (G) Immunoblot for βIII‐tubulin (TUBB3). Actin was used as a loading control. (H) Quantification of the densitometric analysis of βIII‐tubulin band, normalised to actin and expressed relative to the control average. Graph shows means ± SEM of *N* = 3 independent experiments; Statistical analyses were performed for all comparisons by Ordinary one‐way ANOVA; only statistically significant differences are indicated (**p < 0.01; *p < 0.05). (I) Combined Aβ + LSD samples shift towards controls in PCA space defined by Aβ‐altered genes. Principal component analysis was performed on genes significantly altered by Aβ. Each point represents one sample, coloured by treatment group. Marginal density plots show the distribution of samples along PC1 and PC2. Axis labels report the percentage of variance explained by each component. (J, K). Summary scatterplot of GO terms upregulated (J) or downregulated (K) by LSD and oppositely regulated in dementia, showing concordant modulation in genes altered by the addition of LSD to Aβ‐treated neurons. Axes report dementia‐directional and LSD meta‐analysis FDRs, while point colour denotes in vitro significance.

Given that postnatal cortical neuron cultures contain residual astrocytes, we next evaluated astrocyte activation by immunostaining for the astrocytic activation marker GFAP. Aβ treatment induced a significant upregulation of GFAP, which was significantly reduced by subsequent LSD treatment (Figure 3C,D). Again, LSD alone did not affect GFAP expression.

As a proxy of synaptic integrity, we then assessed by immunoblotting the expression of the PSD95 synaptic component in the triton‐insoluble postsynaptic‐enriched fraction. As expected, Aβ exposure reduced PSD95 levels, which were restored to baseline values upon subsequent LSD treatment (Figure 3E,F). Likewise, Aβ treatment induced a significant decrease in the expression of the neuron‐specific βIII‐tubulin isoform that was restored by LSD treatment (Figure 3G,H). Across all four readouts, marker levels in Aβ + LSD‐treated cultures were not significantly different from those of controls, consistent with attenuation towards control levels.

### 2.6 LSD Attenuates Amyloid‐β‐Induced Transcriptional Alterations and Reinstates Neuron‐Associated Gene Expression Programmes

Building on our computational analyses, we investigated whether LSD could reverse Aβ‐induced transcriptional alterations in mouse cortical neurons. To this end, we collected RNA from P2 cortical neurons treated with DMSO (control), Aβ at three different concentrations (100 nM, 500 nM, 1 μM), LSD alone, or a combination of Aβ and LSD. RNA sequencing followed by differential expression analysis showed that the number of DEGs (FDR < 0.05) was reduced in the combined treatment compared with Aβ alone (Figure S13; paired Wilcoxon test *p* = 0.13), suggesting that LSD attenuates the transcriptional impact of Aβ. More strikingly, when restricting the analysis to genes dysregulated by Aβ treatment, principal component analysis revealed a gradient separating control from Aβ‐treated samples, with combined Aβ + LSD samples clustering closer to controls than Aβ‐only samples along both PC1 and PC2 (Figure 3I). Of note, the Aβ‐induced transcriptional signature shows concordance with AD‐associated gene expression changes across 4 out of 5 independent Alzheimer's disease brain datasets (Figure S14), supporting the potential translational relevance of the model.

Notably, Gene Ontology (GO) categories upregulated by LSD in vivo and exhibiting opposite‐direction enrichment as compared to dementia signatures were reduced in primary neurons treated with Aβ and increased by LSD treatment. Among these, the synaptic vesicle maturation and transport category is particularly interesting (Figure 3J, Table S21). Conversely, GO categories associated with gliogenesis and cell–cell adhesion showed consistent downregulation following LSD treatment both in vivo and in vitro, while displaying upregulation across human dementia datasets (Figure 3K).

Collectively, these results show that LSD downregulates glia‐ and adhesion‐associated programmes that increase with ageing and neurodegeneration, while upregulating neuron‐associated programmes that are reduced in these conditions. Thus, LSD modulates these biological processes in the opposite direction to the changes observed during ageing and neurodegeneration.

## 3 Discussion

Our data show that chronic LSD treatment induces gene expression states strongly anti‐correlated with ageing‐associated and, to a lesser extent, dementia‐associated programmes in the prefrontal cortex. This relationship is reproduced across datasets, species and treatment regimens, is significantly stronger compared to a broad panel of pharmacological perturbations, and is accompanied by attenuation of amyloid‐β‐induced structural, molecular and transcriptional alterations in primary cortical cultures.

The strong anti‐correlation observed for LSD relative to 193 CMap compounds argues against a generic property of transcriptional perturbation. However, this feature was not uniformly shared across the available psychoplastogen datasets. Pharmahuasca showed a marked ageing‐opposition profile and the strongest dementia‐opposition signal among the psychoplastogens examined, whereas MDMA displayed a strongly ageing‐concordant profile. These comparisons should nevertheless be interpreted cautiously, because the available datasets differ substantially in dose, treatment regimen and sampling time. Matched experimental designs will therefore be required to determine whether these differences reflect intrinsic pharmacological properties. In particular, whether the contrasting profiles of LSD and MDMA relate to their distinct pharmacology—direct 5‐HT2A agonism versus transporter‐mediated monoamine release—remains to be established.

Gene Ontology analyses provide biological context for the observed transcriptional anti‐correlation. LSD upregulated synaptic and dendritic programmes that typically decline with ageing while downregulating gliogenesis and angiogenesis programmes that increase with age. Related patterns were also observed in dementia datasets. Consistently, in Aβ‐treated cortical cultures, LSD increased programmes that were upregulated by LSD in vivo but suppressed in dementia, while attenuating programmes showing the opposite pattern. This convergence across in vivo LSD treatment, human dementia transcriptomics and an Aβ model supports the relevance of these transcriptional programmes to neurodegeneration‐associated cellular states.

Several limitations should be considered. The human transcriptomic analyses are based on bulk tissue, and age‐related changes in cell‐type composition may contribute to the observed signatures; single‐cell or single‐nucleus data will be required to resolve the cellular origin of these effects. The in vitro model is based on postnatal mouse cortical cultures and therefore does not fully reproduce mature or aged neuronal states, while the measured structural and molecular markers do not establish restoration of neuronal function. Moreover, the three experimental components differ substantially in treatment duration—chronic LSD exposure in rats, acute exposure in mice and 24‐h treatment in vitro—and the acute mouse experiment was sampled at a single 90‐min time point. Whether the transcriptional anti‐correlation persists over time or reflects a transient early response remains unknown.

Despite these limitations, our findings provide a data‐driven rationale for prioritising LSD and related compounds for further investigation in neurodegeneration‐relevant models. More broadly, they extend the transcriptional signature reversion framework to psychedelic pharmacology, suggesting that compounds inducing gene expression states opposite to those associated with ageing and dementia may represent candidates for experimental follow‐up. Our results also generate specific mechanistic hypotheses. Future studies should determine whether the observed transcriptional effects depend primarily on 5‐HT2A–Gq signalling or also involve neurotrophic pathways engaged by LSD, including BDNF–TrkB signalling. Receptor‐selective antagonists, pathway‐specific perturbations, and genetic manipulation of these signalling components could directly test these possibilities and determine whether the ageing‐opposing transcriptional programmes can be dissociated from the broader pharmacological and hallucinogenic effects of LSD.

At the translational level, the synaptic, dendritic, glial and adhesion‐related programmes identified here provide a framework for prioritising compounds that reproduce the ageing‐opposing transcriptional profile without sharing the full pharmacological properties of LSD. These may include non‐hallucinogenic psychoplastogens, modulators of neurotrophic signalling and compounds identified directly through transcriptomic signature screening. The strong dementia‐opposition profile observed for pharmahuasca further highlights the potential of this approach to identify additional candidates for neurodegeneration‐relevant testing. Functional validation in mature neuronal systems and, most importantly, in vivo studies in aged and neurodegeneration models will be required to determine whether these transcriptional and cellular effects translate into measurable cognitive or neuropathological benefit.

To facilitate such prioritisation, all data and results are freely available through an open‐source R Shiny application (https://polilab.shinyapps.io/agereversal/), which allows users to test the brain age‐signature opposition potential of treatments for which transcriptional response signatures are available. The underlying code is available at AuroraSavino90/AgingVsLSD.

## 4 Methods

### 4.1 Ageing Datasets Collection and Curation

We curated human PFC ageing transcriptomic datasets from GEO and GTEx, harmonised gene and metadata annotations, applied dataset‐specific preprocessing and quality filtering, and retained suitable DLPFC/PFC datasets for age‐correlation analyses, as detailed in the Supplementary Methods.

### 4.2 Integration of Ageing Datasets

As dorsolateral prefrontal cortex (DLPFC) samples from healthy subjects were the most represented across our curated ageing datasets (Figure S3C), we focused on this subset of samples for data integration. Particularly, we collated the transcriptomic profiles of 681 DLPFC samples from healthy individuals available across 11 datasets, focusing on a set of 4893 genes shared among these datasets. We observed a pronounced batch effect, which hinged on the association between gene expression and sample age and was reflective of the diverse platforms used to acquire the different datasets (Figure S2A–C).

We identified latent variables with SVA (surrogate variable analysis, removing unwanted variation in high‐throughput experiments; Leek et al. 2012), and removed them from the original data employing a linear model. Following integration, the first principal component of the integrated dataset significantly correlated with subjects' age (Pearson's *R* = 0.60, *p*‐value < 2.2 × 10^−16^; Figure S2D–F).

The effectiveness of the correction was assessed using two complementary approaches. First, we quantified, for each gene, the partial variance explained by age and dataset of origin using nested linear models. Models containing age alone, dataset alone, or both variables were compared through their residual sums of squares; the unique variance associated with each variable was defined as the additional fraction of total expression variance explained when that variable was added to a model already containing the other. Distributions of gene‐wise partial variance explained were compared before and after SVA correction (Figure S4A,B). Second, we quantified residual multivariate dataset structure by predicting dataset of origin from transcriptomic profiles using k‐nearest‐neighbour classification. Classification was performed using the first 20 principal components and *k* = 5, with stratified five‐fold cross‐validation repeated 20 times. Within each fold, principal components were derived exclusively from the training samples, and test samples were projected onto this PC space using the transformation defined by the training data. Identical cross‐validation partitions were used for the uncorrected and SVA‐corrected data. Performance was summarised using balanced accuracy, defined as the mean recall across datasets, to account for differences in sample number across datasets (Figure S4C). We note that the integrated dataset was used to derive the consensus age‐correlation ranking analysed in Figure 1A. All other GSEA‐based analyses were performed per dataset. This design ensures that inter‐dataset batch effects, which SVA correction can reduce but not fully eliminate, do not influence the GSEA results, as each dataset's internal gene ranking is computed from samples processed together under identical conditions.

### 4.3 Psychoplastogens' Datasets

We collected transcriptomics datasets of rodents' PFC treated with psychedelics, entactogens and dissociative compounds (all described as ‘psychoplastogens’). These datasets vary substantially in treatment regimen, dose and post‐treatment sampling timepoint (see Table S5 for details); these differences should be considered when interpreting comparisons across compounds.

For RNA‐seq datasets, we identified differentially expressed genes upon treatment using the DESeq2 R package (Love et al. 2014), while we used the Limma R package (Ritchie et al. 2015) for microarrays (FDR < 0.05). We derived human homologues of mouse and rat genes from HomoloGene (NCBI Resource Coordinators 2016) on the 28/03/2022 for rat genes and the 29/08/2022 for mouse genes.

### 4.4 Selection of Ageing‐Reverting and Ageing‐Concordant Comparators

As controls for interventions known to induce or counteract brain ageing effects, we chose exercise and exposure to an enriched environment (ageing‐reverting), and ethanol administration (ageing‐concordant), deriving signatures of differentially expressed genes (FDR < 0.05) upon intervention from the following GEO datasets: GSE64607 (Hill and Gammie 2022; selecting cortical samples, control vs. voluntary running for 28 days), GSE164798 (Wierczeiko et al. 2021; selecting C57BL/6 mice, control vs. voluntary running for 30 days), GSE111273 (control vs. enriched environment, separately for young and old mice), GSE72507, GSE60676 (Osterndorff‐Kahanek et al. 2015) (selecting prefrontal cortex samples, mice analysed at the end of ethanol/control exposure), GSE28515 (Wolen et al. 2012). As an additional ageing‐concordant comparator, we included signatures of ageing in rats, the model organism used in our primary psychedelics dataset (dataset GSE75772; Ianov et al. 2016).

### 4.5 Identification of Genes Whose Expression Correlates With Age

We used samples from the DLPFC or mPFC of healthy subjects in the curated ageing datasets, discarding datasets with < 10 samples with annotated age ≥ 20 years. We computed Pearson's correlation scores between each gene's expression profile and the pattern of annotated ages across samples.

### 4.6 Testing Ageing‐Signature Opposition Potential

We ranked all the genes based on the correlation of their expression with age (computed as explained above) across samples of the ageing dataset, in decreasing order, that is, with genes whose expression was strongly correlated with age at the top. We used the resulting ranked lists of genes as background ranked lists for a Gene Set Enrichment Analysis (GSEA) (Subramanian et al. 2005) using sets of differentially expressed genes (up‐ or down‐regulated, used separately) upon psychoplastogen‐treatment/exposure‐to‐enriched‐environment/exercise as query signatures. This analysis yielded enrichment scores (ES) with corresponding significance *p*‐values, reflecting the extent to which the genes in the query signatures were ranked consistently across the background lists, that is, down‐regulated genes at the top and up‐regulated genes at the bottom (with large positive ES yielded by down‐regulated genes' query signatures and large negative ES yielded by up‐regulated genes' query signatures indicating strong consistency, thus indicating a potential ageing‐signature opposition pattern).

In this way, we obtained an ageing‐signature opposition *p*‐value for each treatment and each ageing dataset separately for up‐ and down‐regulated genes by the treatment, which we then aggregated across datasets using the Fisher method (Edwards 2005), thus computing a global reliability score for ageing‐signature opposition of each intervention (still keeping up‐ and down‐regulated genes upon treatment separated).

This design ensures that inter‐dataset batch effects do not influence the GSEA results, as each dataset's internal gene ranking is computed from samples processed together under identical conditions. Additionally, the Fisher's method employed is appropriate for aggregating evidence from independent tests and does not assume homogeneity of effect across datasets.

For each intervention and direction of regulation, we summarised the relative evidence for ageing‐signature opposition versus concordance as the ageing‐opposition log *p*‐value ratio (APR):

$$\begin{array}{c} \mathrm{APR}=\log10\left(p\_\text{concordance}/p\_\text{opposition}\right)\\ \;=-\log10\left(p\_\text{opposition}\right)+\log10\left(p\_\text{concordance}\right), \end{array}$$

where p_opposition and p_concordance are the Fisher‐aggregated *p*‐values across ageing datasets under the opposition and concordance hypotheses, respectively. Positive values indicate stronger evidence for opposition than for concordance, negative values the reverse and zero equal evidence. When an aggregated *p*‐value was below the smallest representable floating‐point number (< 2.2 × 10^−308^), the APR was capped at ±300. The APR is a directional summary comparing two hypotheses tested on the same datasets and gene sets, followed by aggregation across datasets and comparison of opposition versus concordance signals, as detailed in the Supplementary Methods. It is not intended as a multiple‐testing correction or an effect‐size measure. The dementia‐opposition ratio was computed analogously.

### 4.7 Comparing the Ageing‐Signature‐Opposition/‐Concordance of Psychoplastogen With That of Ageing‐Concordant and Ageing‐Reverting Comparators

We quantified the ageing‐signature opposition and concordance of each intervention by testing enrichment of treatment‐induced gene sets along ageing‐associated ranked lists using a directional GSEA framework.

### 4.8 Connectivity Map Drug Analysis

We downloaded the Connectivity Map (cMAP) (Subramanian et al. 2017) dataset from Gene Expression Omnibus (GSE70138), from the GSE70138_Broad_LINCS_Level4_ZSPCINF_mlr12k_n345976x12328.gctx file containing the z‐scores resulting from differential expression of each gene (both measured and inferred) between cells treated with a compound and control cells treated with the vehicle, usually DMSO. We considered only perturbations of neural progenitor cells (NPC), as indicated in cMAP's metadata and in case the same perturbation was repeated on multiple samples, we considered the average z‐score for each gene.

For each drug, we defined a drug signature by taking the top differentially expressed genes upon the drug's treatment based on the average z‐score and selecting the same number of genes that we found differentially expressed upon LSD treatment.

We then applied the procedure described in the ‘Testing ageing‐signature opposition potential’ section of the Methods to assess the signature‐opposition of these drugs with ageing (Figures S7 and S8; Figure 1C,D).

### 4.9 Testing Psychoplastogens Dementia‐Signature‐Opposition

We assessed the transcriptional opposition of psychoplastogens to dementia‐associated transcriptional signatures by comparing treatment‐induced gene expression programmes with human PFC dementia datasets using a single‐sample GSEA framework, followed by aggregation and directional comparison of opposition versus concordance effects across datasets, as detailed in the Supplementary Methods.

### 4.10 External Validations

As external validations, we repeated the testing of chronic LSD ageing‐signature opposition defining ageing‐related genes based on additional datasets not found querying GEO but identified through a literature search (GSE25219 and GSE36192). In addition, we tested the dementia‐signature opposition on the Mount Sinai Brain Bank (MSBB) dataset, collecting brain samples from Alzheimer patients, annotated for disease severity. In this case, we selected frontal pole samples from subjects older than 90 years, and ranked the genes based on their correlation with the Braak score (a measure of Alzheimer disease severity) to perform the GSEA on LSD‐regulated genes.

### 4.11 In Vivo Experiments

All experimental procedures were conducted in agreement with the Italian Legislation after approval by the Ministry of Health (E5FC2.N.FK0 and CC652.173.EXT.78). Animals used for this study were kept either at the specific pathogen‐free transgenic unit of the Molecular Biotechnology Center at the University of Turin (for the in vivo LSD treatment experiments) or in standardised hygienic conditions in the Preclinical research facility of Human Technopole in Milan (for the in vitro experiments). The animals had free access to food and water under 12 h/12 h light/dark cycle. All experiments were performed on C57BL/6 mice. In vivo experiments were performed in eight‐week‐old males and the in vitro experiments were performed in P0‐P2 mice, whose sex was not determined as it is not likely to be of relevance for the results obtained in the present study.

Eight‐week‐old male C57BL/6 mice were intraperitoneally injected with either saline (0.9% NaCl) vehicle or LSD at either 50, 100, 200, or 500 μg/kg and euthanized after 90 min through cervical dislocation. The 90‐min time point was chosen to capture the early transcriptional response to acute LSD, consistent with previous studies profiling cortical gene expression 60–90 min after a single psychedelic administration in rodents (Nichols and Sanders‐Bush 2002; González‐Maeso et al. 2003), and to allow comparison with the acute LSD dataset in rat prefrontal cortex included in our analyses. The animals were monitored throughout the treatment period and the treatment was considered effective based on the presence of head‐twitch response (Halberstadt and Geyer 2013). Procedures were conducted at the Molecular Biotechnology Center (University of Turin) in conformity with national and international laws and policies as approved by the Faculty Ethical Committee and the Italian Ministry of Health.

### 4.12 RNA‐Sequencing (In Vivo LSD Treatment)

Brains were removed and sectioned on a vibratome. The range of sections used for analysis corresponds to coronal sections 25–43 of the reference Allen Brain Atlas (Allen Reference Atlas–Mouse Brain [brain atlas]. Available from atlas.brain‐map.org). The PFC areas were isolated and selected following published protocols (Laubach et al. 2018). Total RNA was extracted using Trizol reagent according to manufacturer's instructions.

RNA Integrity was quantified with the TapeStation automated electrophoresis system; standard mRNA stranded Illumina protocols were employed for library preparation, and 40 Million Paired‐end 50 bp reads per sample were sequenced.

We checked the quality of the sequencing with FastQC (https://www.bioinformatics.babraham.ac.uk/projects/fastqc/), aligned the reads to the Grcm38 mouse genome with hisat2 (Kim et al. 2019), and counted the reads aligned to each gene with HTSeq (Anders et al. 2015).

We filtered the genes with at least 10 reads in at least 3 samples, removed the genes with variance < 0.5 after normalising as reads per million, and computed the differentially expressed genes between treated and controls with DESeq2 (Love et al. 2014).

### 4.13 Primary Cortical Neuron Culture

Primary cortical neurons were obtained from 0 to 2 day‐old pups, as described previously (Sclip et al. 2013). Briefly, animals were euthanized and cortex was isolated under a surgical stereomicroscope. Tissues were digested with papain (200 U/mL) in CNDM medium (5.8 mM MgCl2, 0.5 mM CaCl2, 3.2 mM HEPES, 0.2 mM NaOH, 30 mM K2SO4 and 90 mM Na2SO4. pH 7.4, 292 mOsm) supplemented with 0.4% glucose for 30 min at 34°C. Enzymatic activity was blocked with trypsin inhibitors (10 μg/mL, Sigma) in CNDM plus 0.4% glucose for 45 min at room temperature (RT), and the tissue was mechanically dissociated in minimal essential medium (MEM; Invitrogen) supplemented with 10% foetal bovine serum (FBS) (HyperClone) and 0.4% glucose; 0.75 × 105 cells per cm^2^ were plated on poly‐L‐lysine‐coated (0.1 mg/mL) IBIDI (for immunofluorescence) and 35 mm dishes (for RNA extraction and Biochemical analysis). After attachment, the culture medium was switched to Neurobasal‐A (Invitrogen) supplemented with 2% B27‐plus (Invitrogen), 200 mM glutamine and 100 U/mL PenStrep. The medium was changed after 7 days in culture, once a week. No antimitotic agents (e.g., cytosine β‐D‐arabinofuranoside or aphidicolin), were added to suppress glial proliferation, hence the culture contained a residual amount of astrocytes.

The treatments were performed at 18–20 days in vitro (DIV), when neurons are considered to be differentiated. Neurons were pre‐treated with Abeta (Bachem) oligomers at 100 nM for 3 h, as a synaptotoxic stimulus. After a wash‐out, neurons were treated with 10 μM LSD (LGC Standards) for 24 h. This short‐term treatment design, while distinct from the chronic exposure underlying our primary computational analyses, was chosen to model an acute neuroprotective response in a neurodegeneration‐relevant context.

### 4.14 Immunofluorescence

Cortical neurons plated on IBIDI were fixed in 4% paraformaldehyde (PFA) and 4% sucrose in phosphate buffered saline (PBS) for 8 min. For immunofluorescence the following antibodies were used: chicken anti‐MAP2 (Abcam, 1:10,000), rabbit anti‐GFAP (Dako, 1:3000). Secondary antibodies were conjugated with Alexa‐488, Alexa‐555 fluorophores (Invitrogen).

### 4.15 Microscopy and Image Analysis

Images were acquired using Plan Apo Lambda 20×/0.75 NA objective at confocal microscope system Nikon Ti2E inverted microscope equipped with a CrestOptics X‐Light V3 spinning disk unit. Using Fiji ImageJ software the amount of MAP2 and GFAP signal was quantified as the percentage of the analysed area positive for each marker.

### 4.16 Subcellular Fractionation

For the subcellular PSD‐enriched fractionation, cortical neurons plated on 35 mm dish were homogenised in 0.32 M ice‐cold sucrose buffer containing: 1 mM HEPES pH 7.4, 1 mM MgCl2, 1 mM EDTA, 1 mM NaHCO3 and 0.1 mM PMSF, in the presence of a complete set of protease inhibitors (Complete; Roche Diagnostics, Basel, Switzerland) and phosphatase inhibitors (Sigma, St. Louis, MO). Samples were centrifuged at 1000×*g* for 10 min. The resulting supernatant (S1) was centrifuged at 3000×*g* for 15 min to obtain a crude membrane fraction (P2 fraction). The pellet was resuspended in 1 mM HEPES plus protease and phosphatase inhibitor and centrifuged at 100,000×*g* for 1 h at 4°C. The pellet (P3) was resuspended in buffer containing 75 mM KCl and 1% Triton X‐100, incubating 20 min on ice and centrifuged at 100,000×*g* for 1 h at 4°C. The final pellet (P4) referred to as triton‐insoluble postsynaptic fraction (TIF), was resuspended in 20 mM HEPES, sonicated 3 times and stored at −80°C until processing.

### 4.17 Immunoblot

Protein concentrations were quantified using the BCA pierce and 5 μg of TIF extracted proteins were separated by 4%–12% SDS polyacrylamide gel electrophoresis (Invitrogen). Nitrocellulose membranes were blocked in Tris‐buffered saline (5% no fat milk powder, 0.1% Tween20) (1 h, room temperature). Membranes were then incubated overnight at 4°C with rabbit anti‐PSD95 (1:2000, Invitrogen), mouse Beta III tubulin (1:5000, Santa Cruz) and mouse anti‐Actin (1:20000, Millipore), then with HRP‐conjugated secondary antibodies (BioRad) for 1 h at room temperature. Peroxidase activity was detected using ECL (BioRad) and visualised with a ChemiDoc imaging system (BioRad). Image Lab software (Bio‐Rad) was used for quantitative densitometry of protein bands.

### 4.18 RNA‐Sequencing (In Vitro)

RNA was extracted using the Qiagen RNeasy Plus MiNI kit and RNA integrity was measured using an Agilent 4200 Tapestation with an RNA Integrity Number ≥ 9. cDNA libraries were prepared with 150 ng of RNA using the Illumina Stranded mRNA Prep, Ligation Kit (Illumina Inc., San Diego, CA). The libraries were amplified via 14 cycles of PCR and sequenced on a NovaSeq 6000, PE 100, generating an average number of 45 million reads per sample.

Sequences were processed using nf‐core/rnaseq v3.18, with GRCm38 from iGenomes as the reference genome. Briefly, reads were trimmed to remove adapter sequences and low‐quality bases from read ends using Trimgalore v0.6.10, then aligned to the reference genome using STAR v2.6.1d. The resulting alignments were processed with Salmon v1.10.3 for transcriptome quantification. Library reverse strandedness was assessed using RSeQC v5.0.2.

Ensembl IDs were then mapped to gene symbols: when multiple entries mapped to the same symbol, the entry with the highest mean expression across samples was retained. Rows lacking a valid symbol or containing missing symbols were removed.

Principal component analysis (PCA) was performed using FactoMineR (Figure S15).

To mitigate systematic differences across experimental replicates, batch correction was applied to the gene‐level counts matrix using ComBat‐seq (sva package), specifying Replicate as batch. Corrected counts were subsequently converted to library‐size normalised values as reads per million (RPM). PCA was repeated on normalised RPM values to assess the impact of correction (Figure S15). After batch correction, genes were filtered to retain those expressed at ≥ 10 counts in at least 3 samples.

Differential expression was performed with DESeq2. For Aβ dose–response comparisons against control, a DESeqDataSet was constructed from the subset including the controls and the three Aβ concentrations. Separate contrasts were extracted for each Aβ concentration versus control.

Aβ‐responsive genes were defined as the union of significant differentially expressed genes (FDR < 0.05) across the three Aβ concentrations, separated into upregulated (log2FC > 0) and downregulated (log2FC < 0) sets. To evaluate whether LSD attenuates the Aβ transcriptional signature, PCA was performed on RPM values restricted to the union of Aβ‐upregulated and Aβ‐downregulated genes.

To assess whether an Aβ‐induced transcriptional programme observed in vitro is recapitulated in human Alzheimer's disease (AD) brain transcriptomes, we performed per‐dataset differential expression analyses and gene set enrichment testing across multiple independent cohorts (Table S11). Differential expression between AD and control samples was computed independently for each dataset using limma. Genes were ranked by the differential expression log2 fold change (AD vs. control), and enrichment was tested separately for the Aβ up and down gene sets after mapping mouse gene identifiers to human orthologs using biomart.

### 4.19 Functional Enrichment Analysis

We performed Gene Ontology enrichment analyses across human ageing and dementia datasets, in vivo LSD perturbations and in vitro Aβ/LSD‐treated neurons to identify biological processes associated with age‐ and dementia‐related transcriptional changes and to assess their modulation by LSD, integrating results across datasets and testing for concordant or opposing regulation, as detailed in the Supplementary Methods.

### 4.20 ShinyApp

We built a web tool to test the age transcriptional‐opposition potential of a set of user‐defined genes. Users can select the conditions to compute the relationship between gene expression and age, setting the age range, gender, ethnicity, health status and brain region: the same human brain transcriptomic data employed in this work are used for this analysis.

For a given gene set, the tool produces plots equivalent to Figure 1B,E, following the procedures described in the sections ‘Testing ageing‐signature opposition potential’ and ‘Comparing the ageing‐signature‐opposition/‐concordance potential of psychoplastogens with that of ageing‐concordant and ageing‐reverting comparators’. It also reports the APR (defined above), rescaled to the interval [−1, 1] for ease of interpretation, where 1 indicates maximum age opposition while −1 indicates maximum ageing concordance. The tool is freely accessible at the link https://polilab.shinyapps.io/agereversal/.

### 4.21 Code Availability

The code to reproduce the analyses and generate the figures/tables presented in this work is deposited at https://github.com/AuroraSavino90/AgingVsLSD. R and package versions are indicated in File S2.

### 4.22 Declaration of Generative AI and AI‐Assisted Technologies in the Writing Process

During the preparation of this work, the authors used ChatGPT (OpenAI) to assist with language editing and proofreading, with the aim of improving clarity, grammar and readability. No content generation, data analysis, or scientific interpretation was performed by the tool. All authors reviewed, revised and approved the final version of the manuscript and take full responsibility for its content.

## Author Contributions

A.S. and F.I. designed the study and wrote the original draft of the manuscript. A.S. performed the computational analyses. C.L, S.R., M.C. performed in vivo LSD treatments and collected brain samples. G.R.M. and L.A. supervised the in vivo experiments. I.B. performed in vitro experiments on primary cortical neurons, immunofluorescence and immunoblots. F.I., N.K. and V.P. supervised the project. All authors reviewed, revised and approved the final version of the manuscript.

## Funding

Research in the Kalebic lab is supported by grants from AIRC (MFAG 2022 ID 27157) and Gilbert Family Foundation (#923004 and #925001) to N.K. Research in the Poli lab was supported by the Italian Cancer Research Association (AIRC IG24851 to V.P.); the Italian Ministry of University and Research (MUR PRIN 2022BFF2CJ to V.P.), and the National Center for Gene Therapy and Drugs based on RNA Technology, Spoke2, PNRR M4C2‐Investimento 1.4‐CN00000041‐VP.

## Conflicts of Interest

F.I. receives funding from Open Targets, a public‐private initiative involving academia and industry, and from Nerviano Medical Sciences, performs consultancy for CoSyne Therapeutics and is a member of the scientific advisory board of Drug ReKindle. All other authors declare no competing interests.

## Supporting information


**Figure S1:** Samples and their annotations across the ageing transcriptomic datasets.
**Figure S2:** Integration and batch correction of 11 transcriptomic DLPFC datasets.
**Figure S3:** Similarity and distribution of gene–age correlations across ageing datasets and sample composition across annotated categories.
**Figure S4:** SVA correction reduces dataset‐associated variation in the integrated DLPFC dataset.
**Figure S5:** Distribution of normalised enrichment scores across human ageing datasets for each intervention.
**Figure S6:** Concordance of curated ageing datasets with an independent published brain ageing signature.
**Figure S7:** Distribution of ageing‐opposition GSEA *p*‐values for Connectivity Map compounds based on drug‐upregulated genes.
**Figure S8:** Distribution of ageing‐opposition GSEA *p*‐values for Connectivity Map compounds based on drug‐downregulated genes.
**Figure S9:** Cumulative transcriptional opposition (CTO) of intervention‐induced gene expression signatures to ageing‐ and dementia‐associated transcriptional changes.
**Figure S10:** Distribution of Cohen's d values across dementia datasets for genes up‐regulated or down‐regulated by each intervention.
**Figure S11:** External validation of chronic LSD transcriptional opposition using independent ageing datasets and the Mount Sinai Brain Bank cohort.
**Figure S12:** Top 20 Gene Ontology (GO) categories statistically enriched in the genes up‐regulated by LSD and down‐regulated in dementia, and in the genes down‐regulated by LSD and up‐regulated in dementia.
**Figure S13:** Number of significantly differentially expressed genes relative to control for Aβ alone and for the corresponding combined Aβ + LSD treatment across three Aβ concentrations (100 nM, 500 nM, 1 μM).
**Figure S14:** Gene set enrichment analysis of the Aβ‐induced transcriptional signature across five human Alzheimer's disease brain datasets.
**Figure S15:** Principal component analysis of in vitro RNA‐seq samples.
**Figure S16:** Correcting a normalisation bias introduced by mitochondrial genes in the GTEx dataset.
**Table S1:** Ageing datasets retrieved by the search strategy described in the Supplementary Methods but excluded from the analysis.
**Table S2:** Ageing transcriptomic datasets included in the analysis.
**Table S3:** Number of samples of the ageing datasets considered in our analysis across annotated features.
**Table S5:** Psychoplastogen datasets included in the analysis.
**Table S6:** Ageing‐Reverting and Ageing‐Concordant Comparator datasets included in the analysis, with corresponding labels used in Figure 1EF.
**Table S11:** Dementia datasets.

**Data S1:** Editable Supp Figure Captions.

**Table S4:** GSEA results for each combination of ageing dataset, intervention dataset and direction of differential expression.

**Table S7:** Meta‐analysed *p*‐values and ageing‐opposition log *p*‐value ratios (APR) for each intervention.

**Table S8:** Mean expression change of intervention‐regulated genes used in the ageing and dementia analyses.

**Table S9:** Cumulative transcriptional opposition scores of intervention‐induced gene expression signatures relative to ageing‐ and dementia‐associated transcriptional changes.

**Table S10:** Intervention‐regulated genes changing opposite to or concordantly with human brain ageing signatures, per reference dataset.

**Table S12:** GSEA results for all combinations of dementia dataset, intervention dataset and direction of differential expression.

**Table S13:** Meta‐analysed *p*‐values and dementia‐opposition *p*‐value ratios for each intervention.

**Table S14:** Intervention‐regulated genes changing opposite to or concordantly with dementia signatures, per reference dataset.

**Table S15:** GSEA results for each combination of ageing dataset, LSD concentration and direction of differential expression.

**Table S16:** Meta‐analysed *p*‐values and ageing‐opposition *p*‐value ratios for each LSD concentration.

**Table S17:** Functional Enrichment for genes up‐regulated by LSD and down‐regulated upon ageing.

**Table S18:** Functional Enrichment for genes down‐regulated by LSD and up‐regulated upon ageing.

**Table S19:** Functional Enrichment for genes up‐regulated by LSD and down‐regulated in dementia.

**Table S20:** Functional Enrichment for genes down‐regulated by LSD and up‐regulated in dementia.

**Table S21:** Full list of Gene Ontology categories tested for differential expression in primary neurons treated with LSD plus Aβ relative to Aβ alone.

**File S1:** GEO search results for the 95 raw transcriptomic datasets retrieved by the ageing‐dataset search strategy.

**File S2:** R and package versions used for the analyses.

## Data Availability

RNA‐sequencing data generated in this work were deposited in the Gene Expression Omnibus and are accessible at https://www.ncbi.nlm.nih.gov/geo/ under the accession numbers GSE263662 and GSE326966. All the other data are publicly available and accessible as detailed in the Section 4 and the Supplementary Methods.
