# Supplementary material for: Lysergic Acid Diethylamide Induces Transcriptional Changes Opposing Signatures of Brain Ageing and Neurodegeneration
Source: Aging Cell. 2026 Nov 10;25(11):e70793. doi: 10.1111/acel.70793 (PMC13751324; doi:10.1111/acel.70793)
Supplement: Supplementary file 1 [file ACEL-25-e70793-s010.pdf]

Supplementary Material (Tables, Figures and Methods) for

Lysergic acid diethylamide induces transcriptional changes opposing signatures of brain aging and neurodegeneration

Aurora Savino[1,*], Carla Liaci[2], Ilaria Bertani[1], Simona Rando[2], Mattia Camera[2], Giorgio R. Merlo[2], Lidia Avalle[2,3], Valeria Poli[2,+], Nereo Kalebic[1,+], Francesco Iorio[1,+,*]

[1] Human Technopole, Computational Biology, Palazzo Italia, Viale Rita Levi Montalcini 1, 20157 Milan, Italy
[2] Molecular Biotechnology Center, Department of Molecular Biotechnology and Health Sciences, University of Turin, via Nizza 52, Turin 10126, Italy
[3] Department of Science and Technological Innovation, University of Piemonte Orientale, Viale Teresa Michel 11, 15121 Alessandria, Italy
+ co-senior authors; * correspondence: aurora.savino@unito.it and francesco.iorio@fht.org

Supplementary Figures

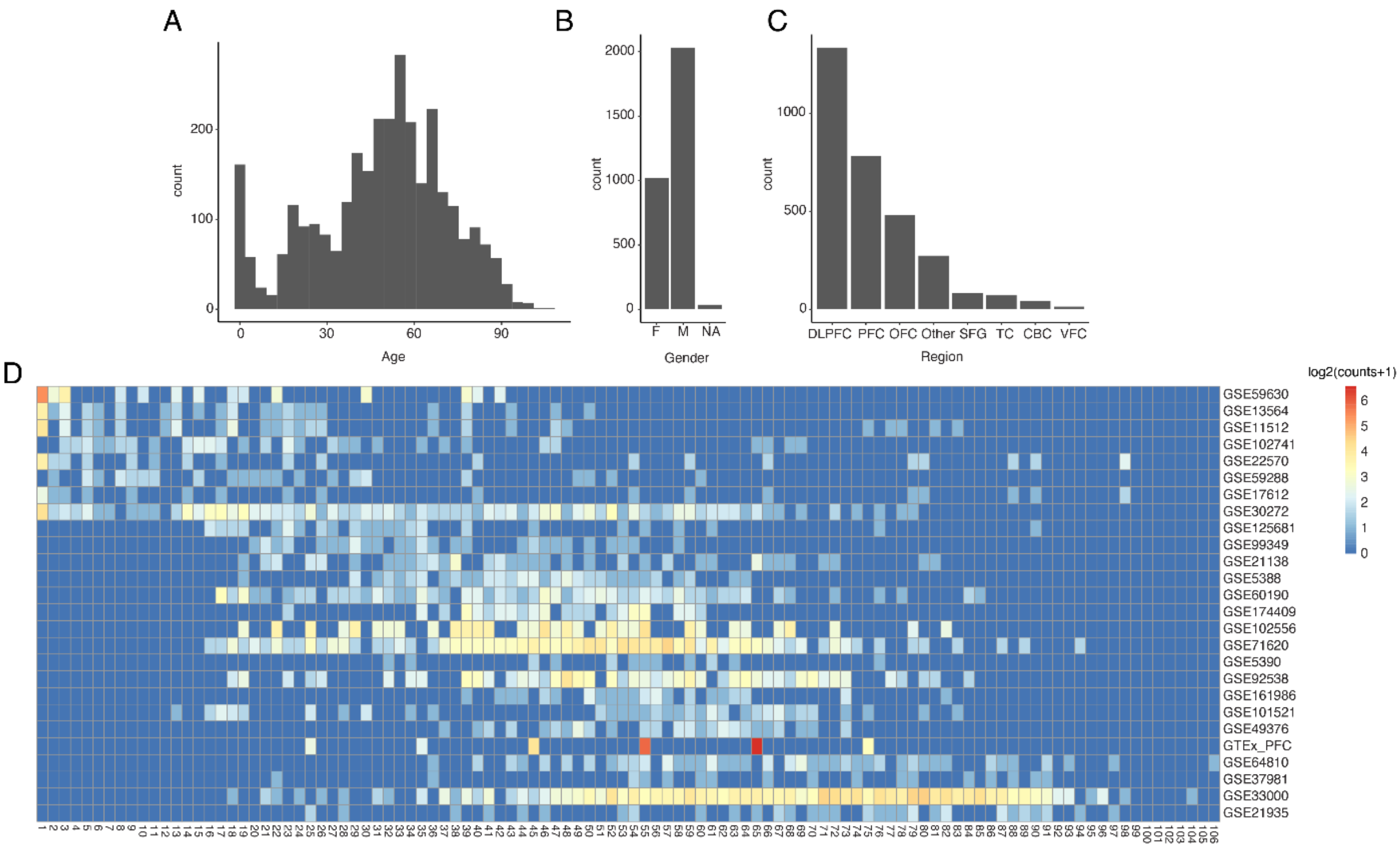

Figure S1. Samples and their annotations across the aging transcriptomic datasets.

A. Distribution of sample age (years). **B.** Distribution of gender (M = Male, F = Female, NA = Not available). **C.** Distribution of sampled brain regions (DLPFC = dorsolateral prefrontal cortex; PFC = prefrontal cortex; OFC = orbitofrontal cortex; SFG = superior frontal gyrus; TC = temporal cortex; CBC = cerebellar cortex; VFC = ventral frontal cortex). **D.** Number of samples by age (columns) within each dataset (rows).

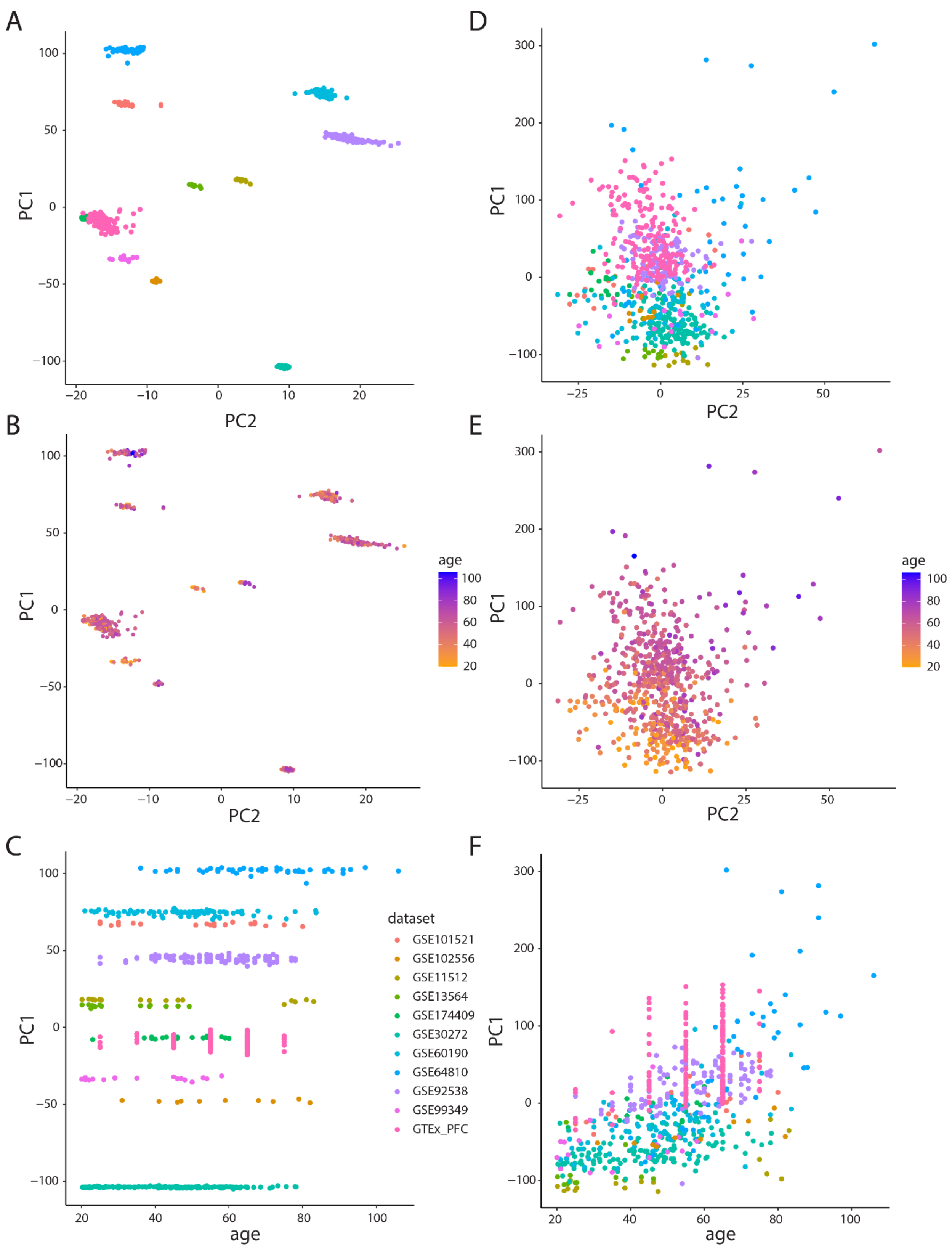

Figure S2. Integration and batch correction of 11 transcriptomic DLPFC datasets. A-C. Principal component analysis before batch correction. **A.** Samples projected onto the first two principal components (PC1 and PC2), coloured by dataset of origin, showing a strong dataset-associated batch effect. **B.** The same PCA projection, with samples coloured according to subject age. **C.** Relationship between subject age and PC1 before correction, showing little association between

the principal source of transcriptional variation and age. **D-F.** Corresponding analyses after batch correction. **D.** Samples coloured by dataset of origin show substantially reduced dataset-specific clustering. **E.** PCA projection coloured according to subject age. **F.** Relationship between subject age and PC1 after correction, revealing a significant positive correlation (Pearson's $R = 0.60$, $p < 2.2 \times 10^{-16}$).

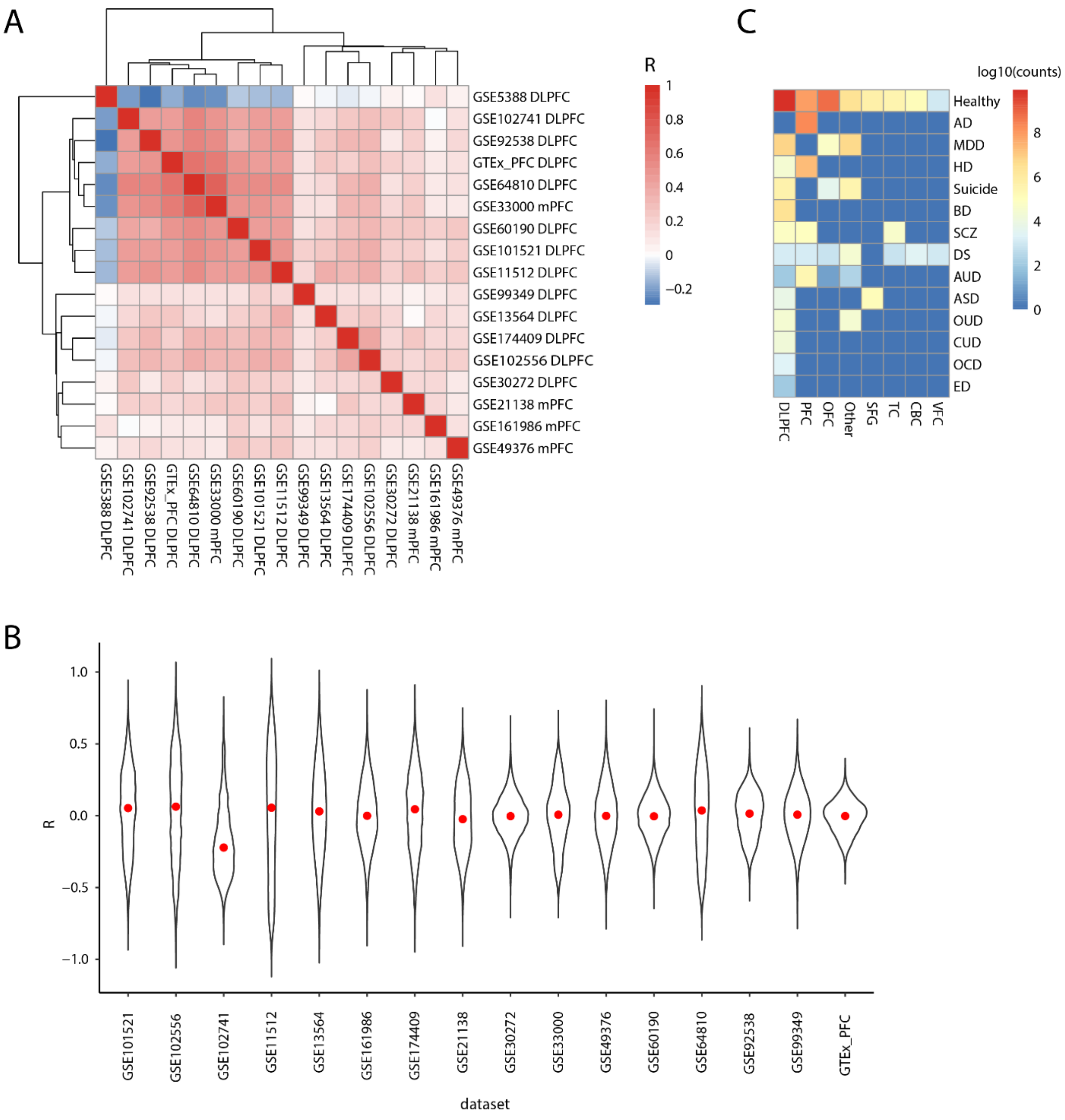

Figure S3. A. Similarity (Pearson's correlation) between genes' correlation with age in different ageing datasets. B. Distribution of genes' correlation with age in different ageing datasets (median in red). C. Number of samples in the ageing datasets collection belonging to each combination of categories. DLPFC = dorsolateral prefrontal cortex; mPFC = medial prefrontal cortex; OFC = orbitofrontal cortex; SFG = superior frontal gyrus; TC = temporal cortex, CBC = cerebellar cortex; VFC = ventral frontal cortex; AD = Alzheimer disease; MDD = major depressive disorder; HD = Huntington disease; BD = bipolar disorder; SCZ = schizophrenia; DS = Down syndrome; AUD = alcohol use disorder; ASD = autism spectrum disorder; OUD = opioid use disorder; CUD = cannabis use disorder; OCD = obsessive compulsive disorder; ED = eating disorder.

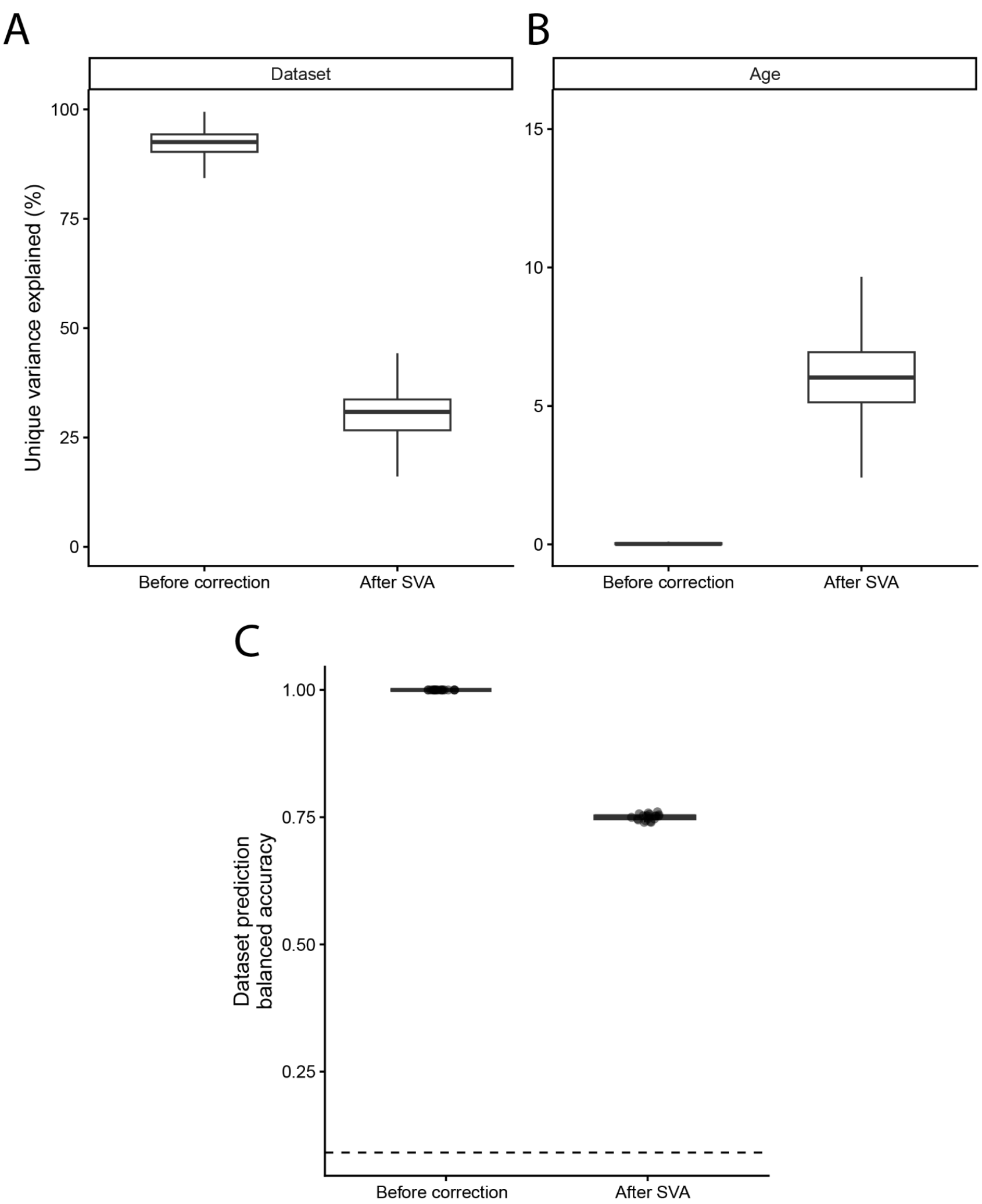

Figure S4. SVA correction reduces dataset-associated variation in the integrated DLPFC dataset. A. Distribution across 4,893 genes of the fraction of expression variance uniquely associated with the dataset of origin. B. Distribution across the same genes of the fraction of expression variance uniquely associated with age after accounting for dataset of origin. C. Residual dataset-specific structure assessed by K-nearest-neighbour classification of dataset of origin. Points represent mean balanced accuracy across folds for each of 20 repeated cross-validation runs; the dashed line indicates chance-level balanced accuracy (1/11).

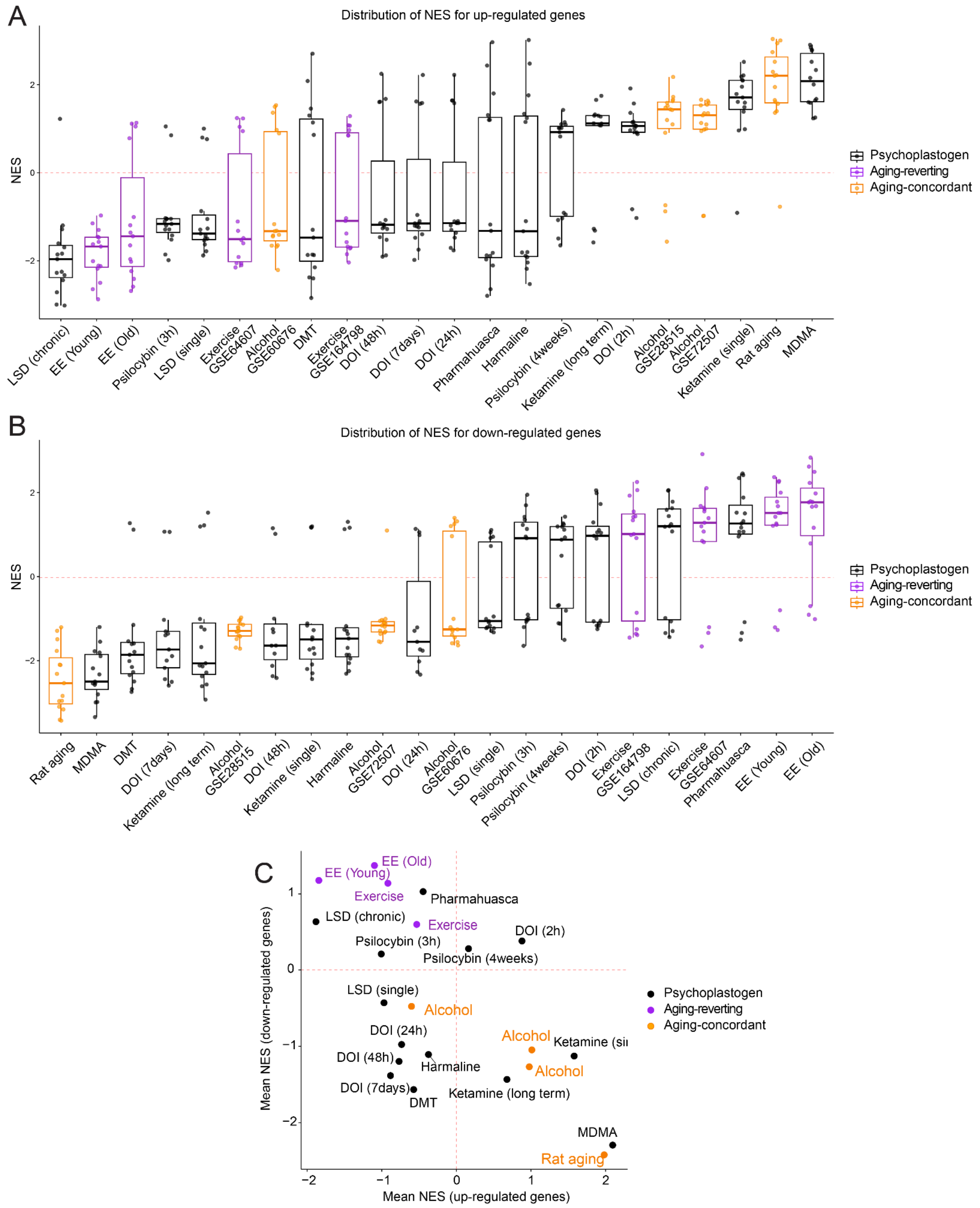

Figure S5. Distribution of normalized enrichment scores across human aging datasets for each intervention. A, **B**. Distribution of normalized enrichment scores (NES) across human aging datasets for genes upregulated (**A**) or downregulated (**B**) by each intervention. Each point represents one aging dataset, and boxplots summarize the distribution across datasets. Interventions are ordered by mean NES within each panel. Colours denote intervention class: psychoplastogens in black, aging-reverting comparators in purple, and aging-concordant comparators in orange. C. Mean NES for intervention-upregulated genes plotted against mean NES for intervention-downregulated genes across aging datasets. Interventions in the upper-left quadrant display the strongest aging-opposing pattern, with upregulated genes enriched among genes that decrease with age (negative NES) and downregulated genes enriched among genes that increase with age (positive NES).

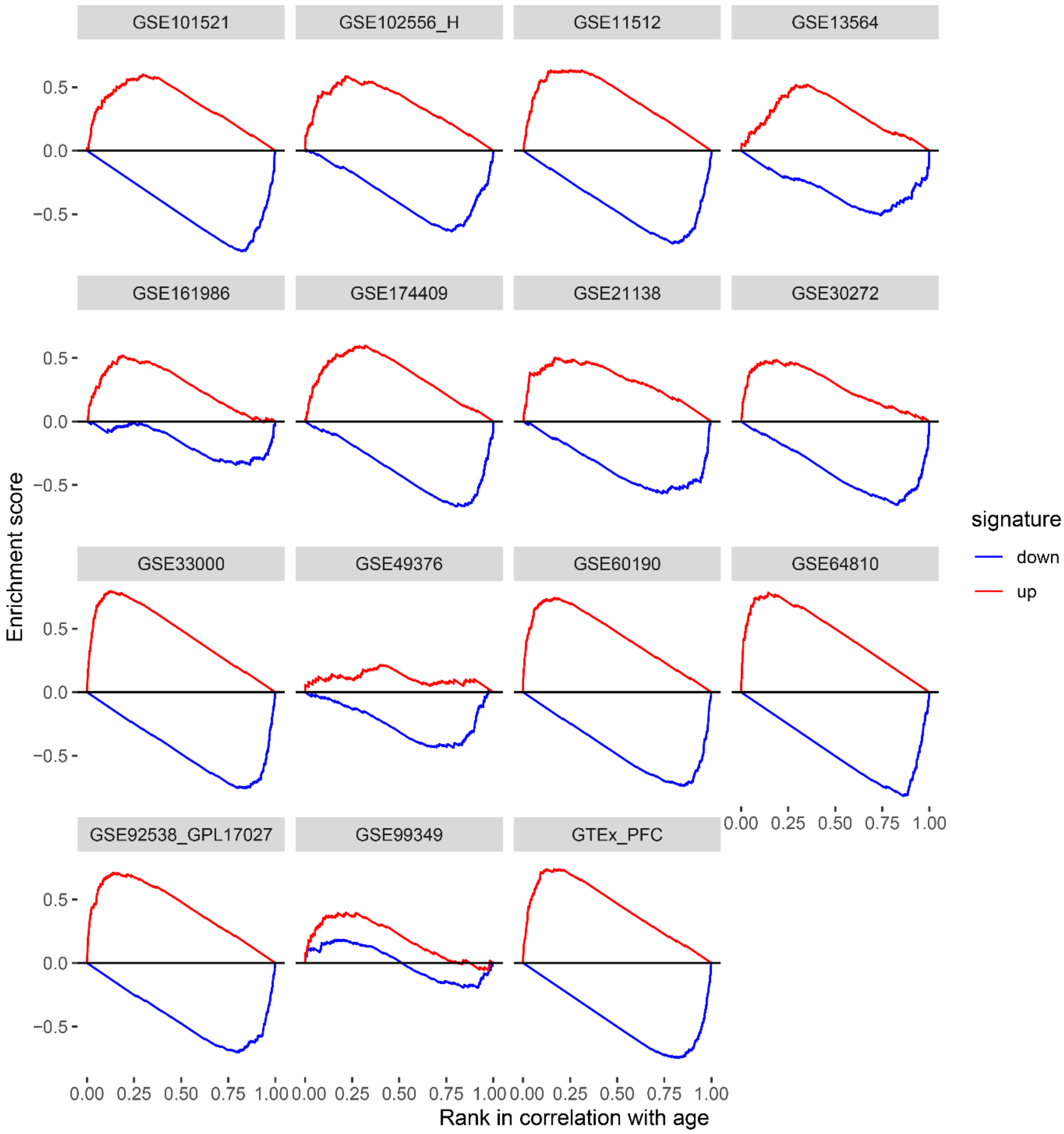

Figure S6. Concordance of curated aging datasets with an independent published brain aging signature. Gene Set Enrichment Analysis (GSEA) performed separately for each dorsolateral or medial prefrontal cortex aging dataset, with genes ranked according to the correlation between their expression and sample-age. Genes reported by Dönertaş et al. (Dönertaş et al. 2018) to increase or decrease in expression with age were used as separate query signatures. Red and blue curves indicate enrichment of age-upregulated and age-downregulated genes, respectively.

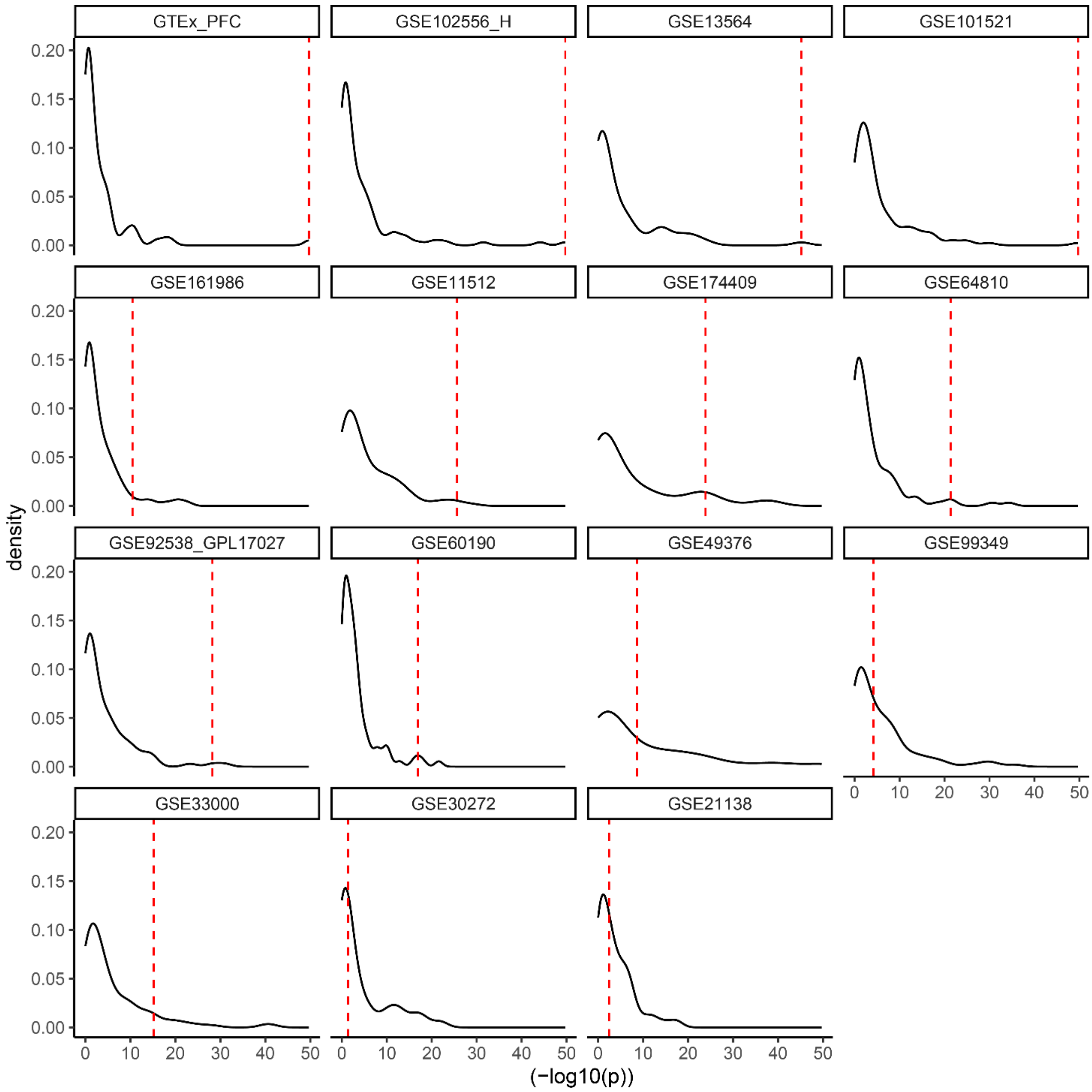

Figure S7. Distribution of aging-opposition GSEA p-values for Connectivity Map compounds based on drug-upregulated genes. For each human aging dataset, the black density curve shows the distribution of GSEA p-values obtained for compounds profiled in neural progenitor cells in the Connectivity Map, using genes upregulated by each compound as query signatures. P-values are shown as -log10(p). The dashed red line indicates the corresponding value obtained for the chronic LSD transcriptional signature.

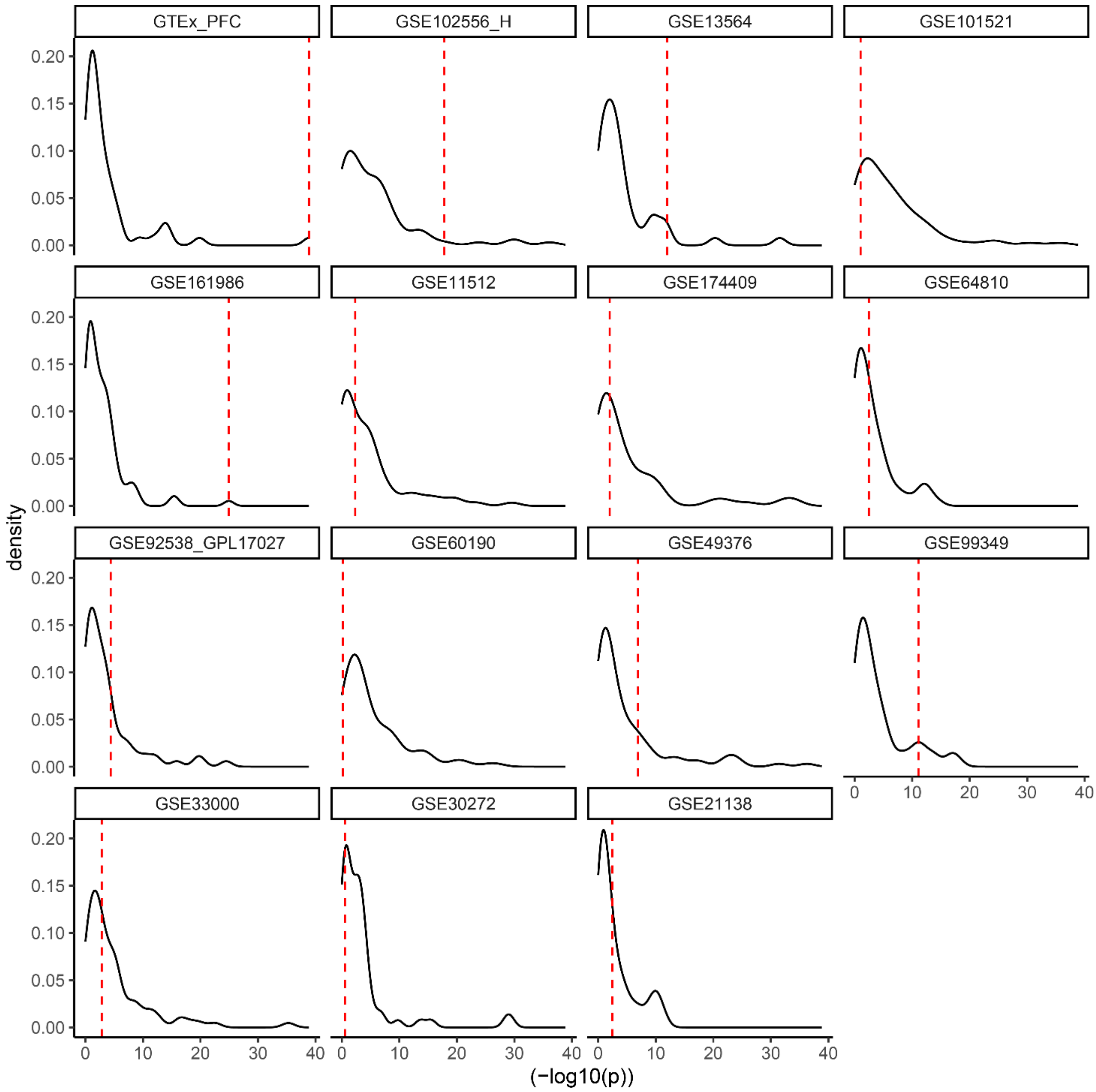

Figure S8. Distribution of aging-opposition GSEA p-values for Connectivity Map compounds based on drug-downregulated genes. For each human aging dataset, the black density curve shows the distribution of GSEA p-values obtained for compounds profiled in neural progenitor cells in the Connectivity Map, using genes downregulated by each compound as query signatures. P-values are shown as -log10(p). The dashed red line indicates the corresponding value obtained for the chronic LSD transcriptional signature.

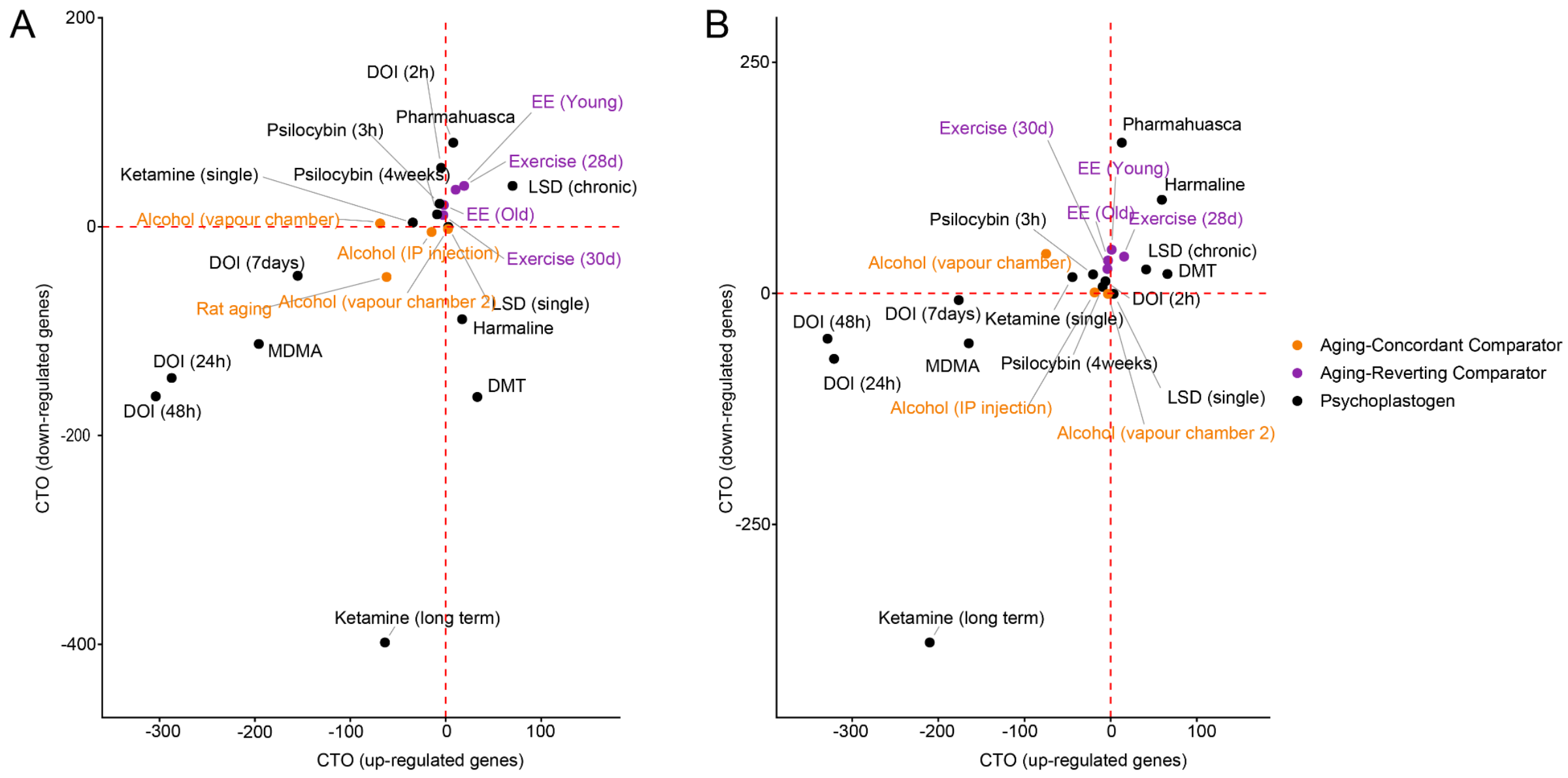

Figure S9. Cumulative transcriptional opposition (CTO) of intervention-induced gene expression signatures to aging- and dementia-associated transcriptional changes. A. CTO relative to human brain aging signatures. B. CTO relative to dementia-associated transcriptional signatures. For each intervention and reference dataset, genes were classified as opposing or concordant according to the direction of the intervention-induced logFC relative to the corresponding aging- or dementia-associated expression change. CTO was calculated separately for intervention-upregulated (x-axis) and intervention-downregulated (y-axis) genes. Red dashed lines indicate zero. EE = enriched environment.

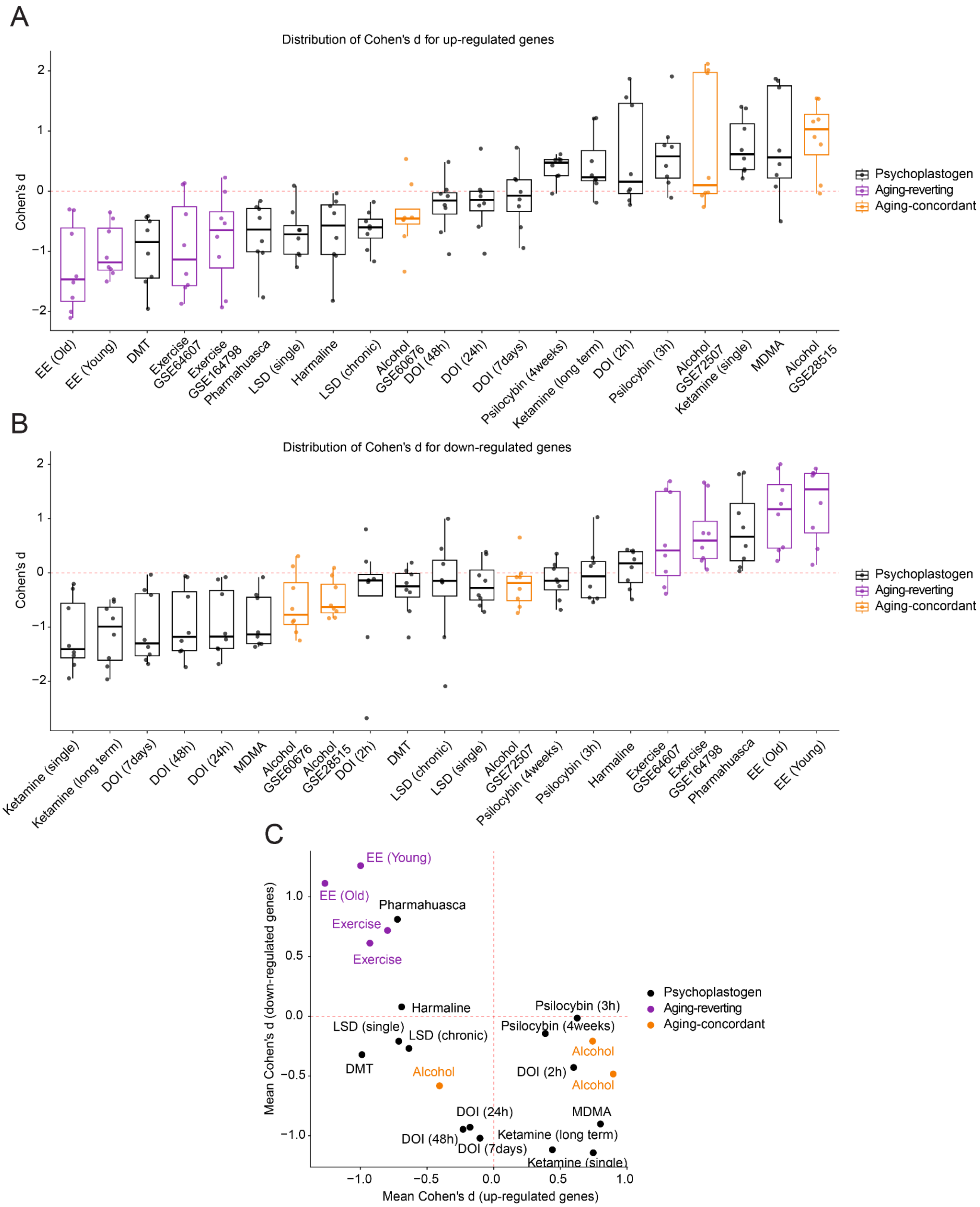
A
Distribution of Cohen's d for up-regulated genes
Cohen's d
Psychoplastogen
Aging-reverting
Aging-concordant
EE (Old)
EE (Young)
DMT
Exercise GSE64607
Exercise GSE164798
Pharmahuasca
LSD (single)
Harmaline
LSD (chronic)
Alcohol GSE60676
DOI (48h)
DOI (24h)
DOI (7days)
Psilocybin (4weeks)
Ketamine (long term)
DOI (2h)
Psilocybin (3h)
Alcohol GSE72507
Ketamine (single)
MDMA
Alcohol GSE28515
B
Distribution of Cohen's d for down-regulated genes
Cohen's d
Psychoplastogen
Aging-reverting
Aging-concordant
Ketamine (single)
Ketamine (long term)
DOI (7days)
DOI (48h)
DOI (24h)
MDMA
Alcohol GSE60676
Alcohol GSE28515
DOI (2h)
DMT
LSD (chronic)
LSD (single)
Alcohol GSE72507
Psilocybin (4weeks)
Psilocybin (3h)
Harmaline
Exercise GSE64607
Exercise GSE164798
Pharmahuasca
EE (Old)
EE (Young)
C
EE (Young)
EE (Old)
Pharmahuasca
Exercise
Exercise
Harmaline
Psilocybin (3h)
LSD (single)
LSD (chronic)
Psilocybin (4weeks)
DMT
Alcohol
Alcohol
DOI (2h)
Alcohol
DOI (24h)
MDMA
DOI (48h)
Ketamine (long term)
DOI (7days)
Ketamine (single)
Mean Cohen's d (down-regulated genes)
Mean Cohen's d (up-regulated genes)
Psychoplastogen
Aging-reverting
Aging-concordant

Figure S10. A, B. Distribution of Cohen's d values across dementia datasets for genes up-regulated (A) or down-regulated (B) by each intervention. Each point represents one dementia dataset/condition comparison, and boxplots summarize the distribution across datasets. Interventions are ordered by median Cohen's d within each panel. Colors indicate the global signature class: psychoplastogens in black, aging-reverting interventions in purple, and aging-concordant comparators in orange. C. Mean Cohen's d for up-regulated intervention genes versus mean Cohen's d for down-regulated intervention genes across dementia datasets. Points represent interventions, colored according to the same signature classes as in panels (A,B). This summary highlights the relative positioning of psychoplastogens, aging-reverting comparators, and aging-concordant comparators with respect to dementia-associated transcriptional changes.

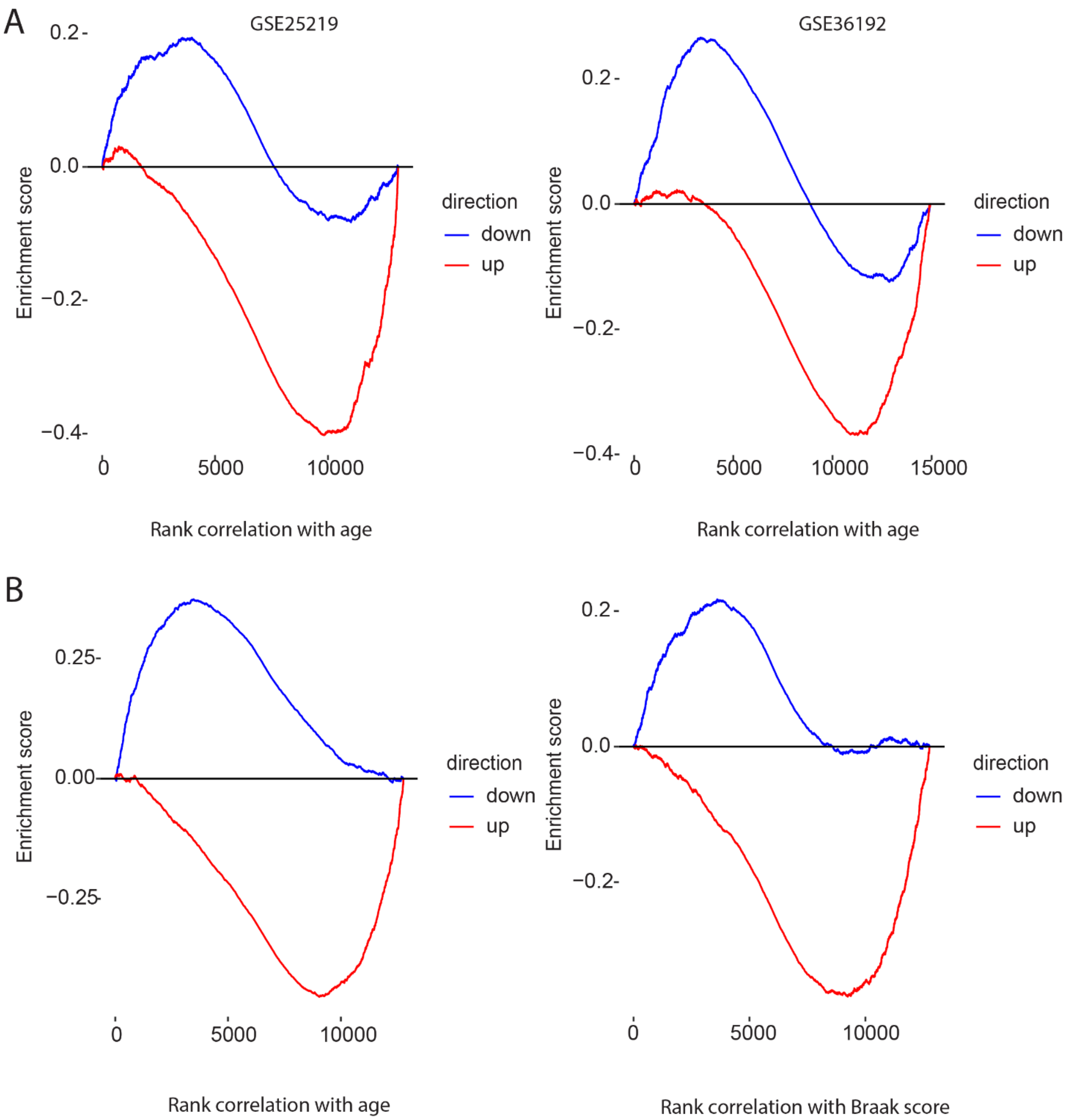

Figure S11. A. Gene set enrichment analysis of genes up- and down-regulated upon chronic LSD in rats (used as queries) along a background ranked list of genes sorted based on the correlation between their expression and sampled subjects' age in two additional datasets not included in the main analysis, used as external validations**.** B. Gene set enrichment analysis of genes up- and down-regulated upon chronic LSD in rats for genes with high correlation with age according to the Mount Sinai Brain Bank (left) and for genes with high correlation with the Braak score of Alzheimer's disease severity in the same dataset (right).

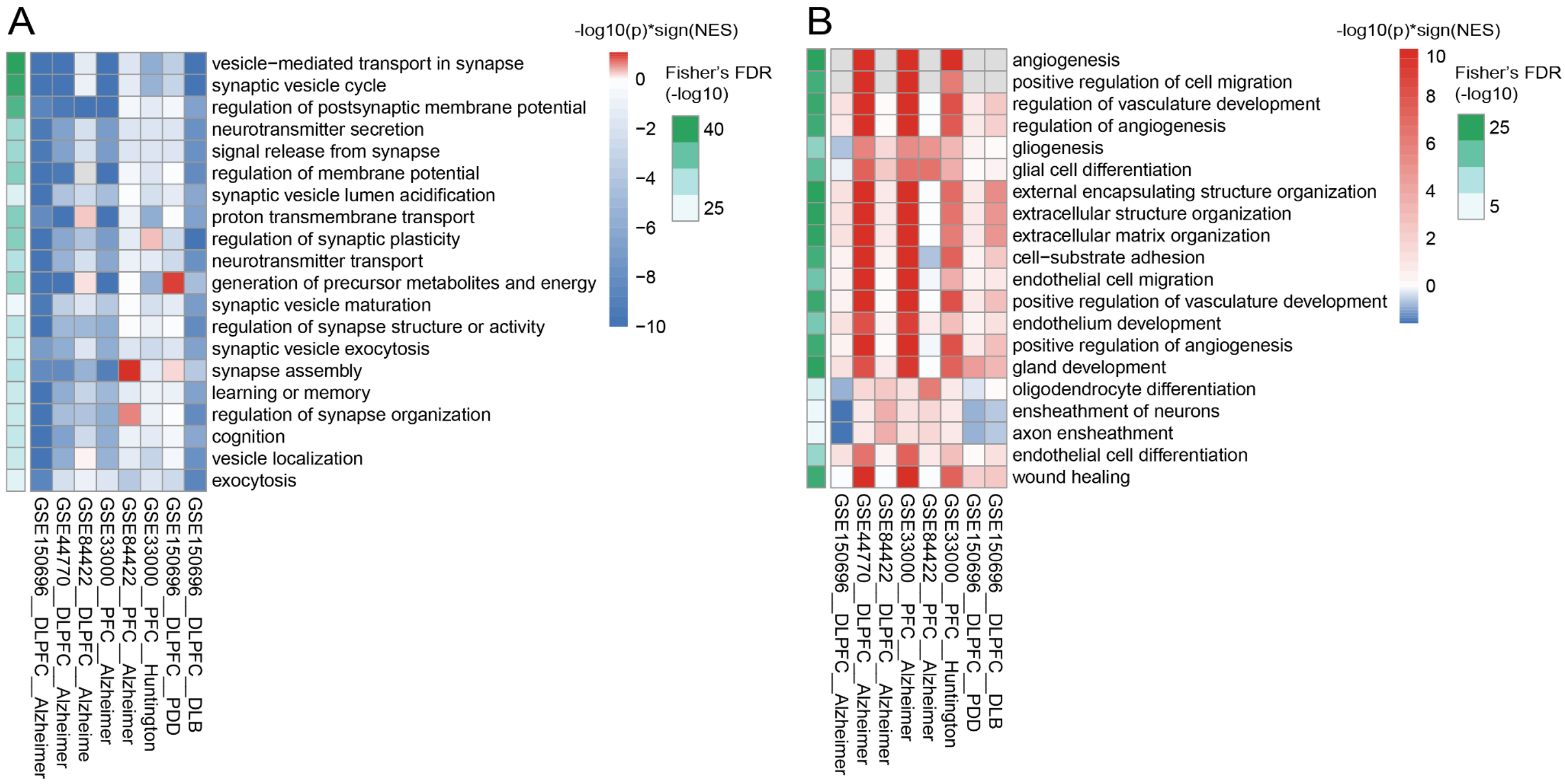

Figure S12. Top 20 Gene Ontology (GO) categories statistically enriched in the genes up-regulated by LSD and down-regulated in dementia (A), and in the genes down-regulated by LSD and up-regulated in dementia (B). The colour in the heatmap indicates the GSEA p-value (-log10) with sign depending on the sign of the normalized enrichment score (NES). Red, highly significant positive enrichments; blue, highly significant negative enrichments. The green bar on the left indicates the overall FDR (-log10) combining the p-values obtained for each dataset.

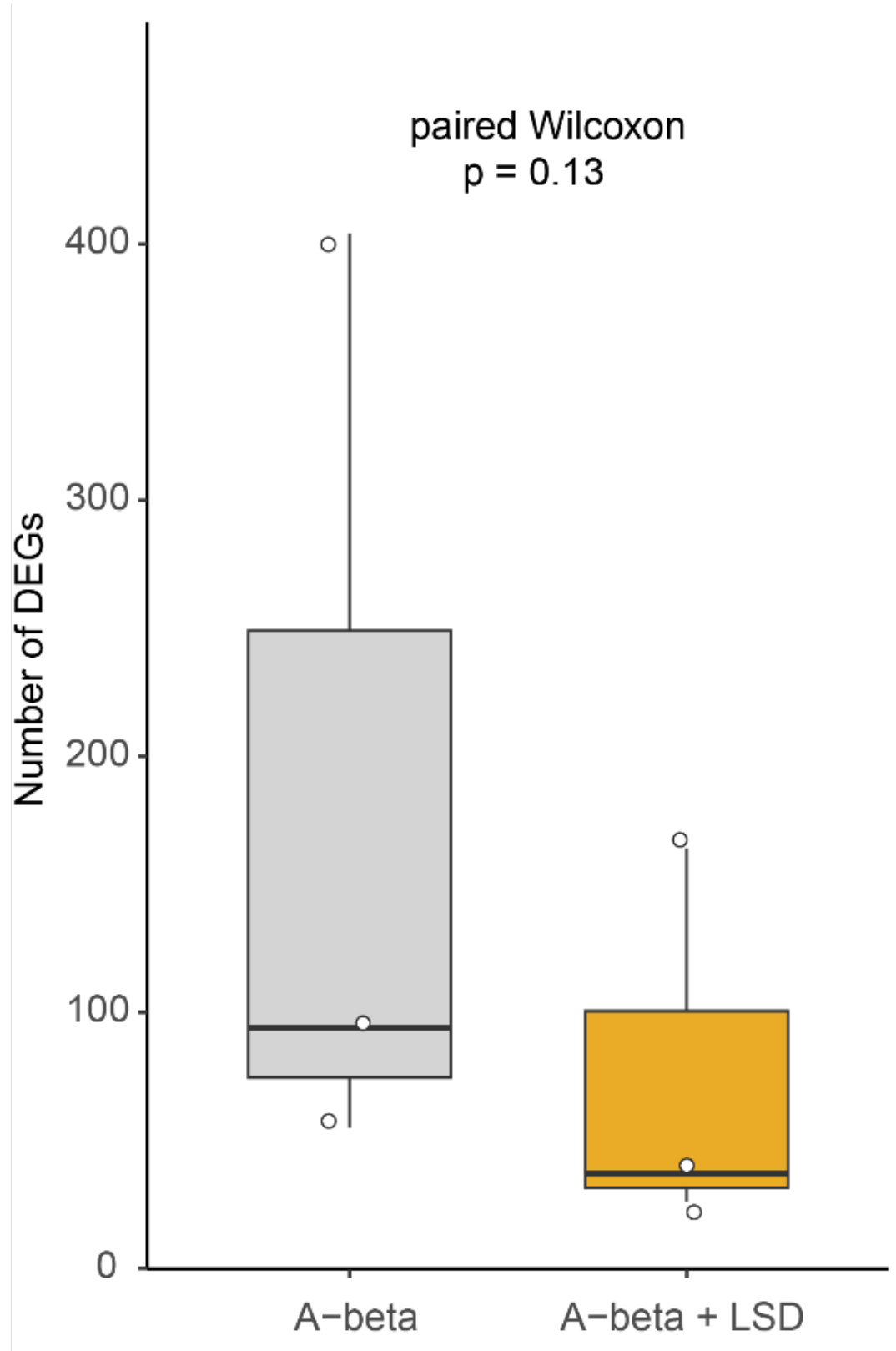

Figure S13. Number of significantly differentially expressed genes (DEGs; FDR < 0.05) relative to control for Aβ alone and for the corresponding combined Aβ+LSD treatment across three Aβ concentrations (100 nM, 500 nM, 1 µM). The p-value was computed using a paired Wilcoxon signed-rank test (one-sided), pairing each Aβ dose with its matched Aβ+LSD condition.

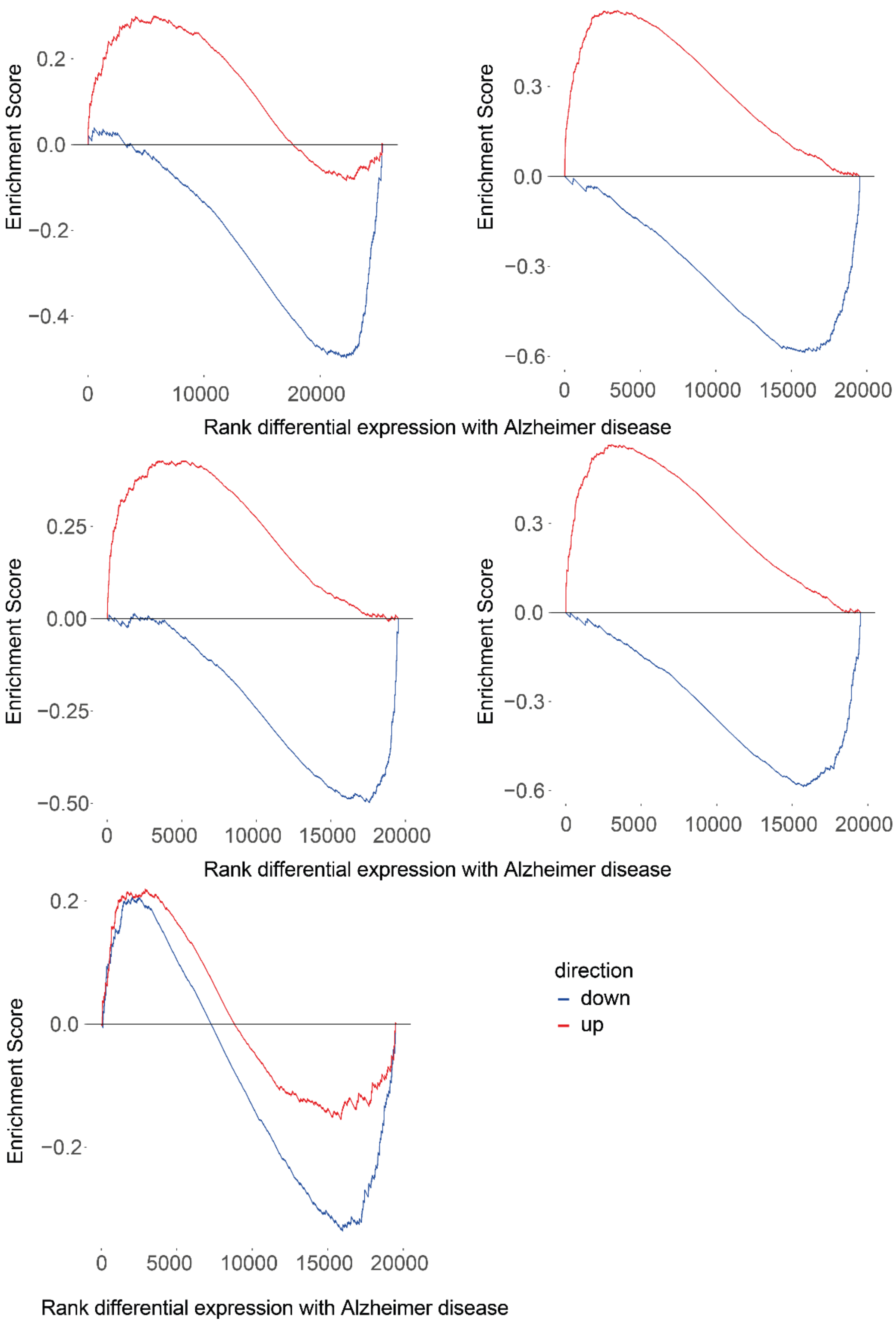

Figure S14. Gene set enrichment analysis (GSEA) was performed independently for five human brain datasets comparing Alzheimer's disease (AD) versus healthy control samples. Genes were ranked by log2 fold change differential expression analysis (AD vs control). Enrichment curves show the running enrichment score for genes upregulated (red) or downregulated (blue) by Aβ treatment in vitro, after mapping to human orthologs. Positive enrichment indicates concordance between the in vitro Aβ signature and transcriptional changes observed in AD brain tissue, whereas negative enrichment indicates inverse regulation.

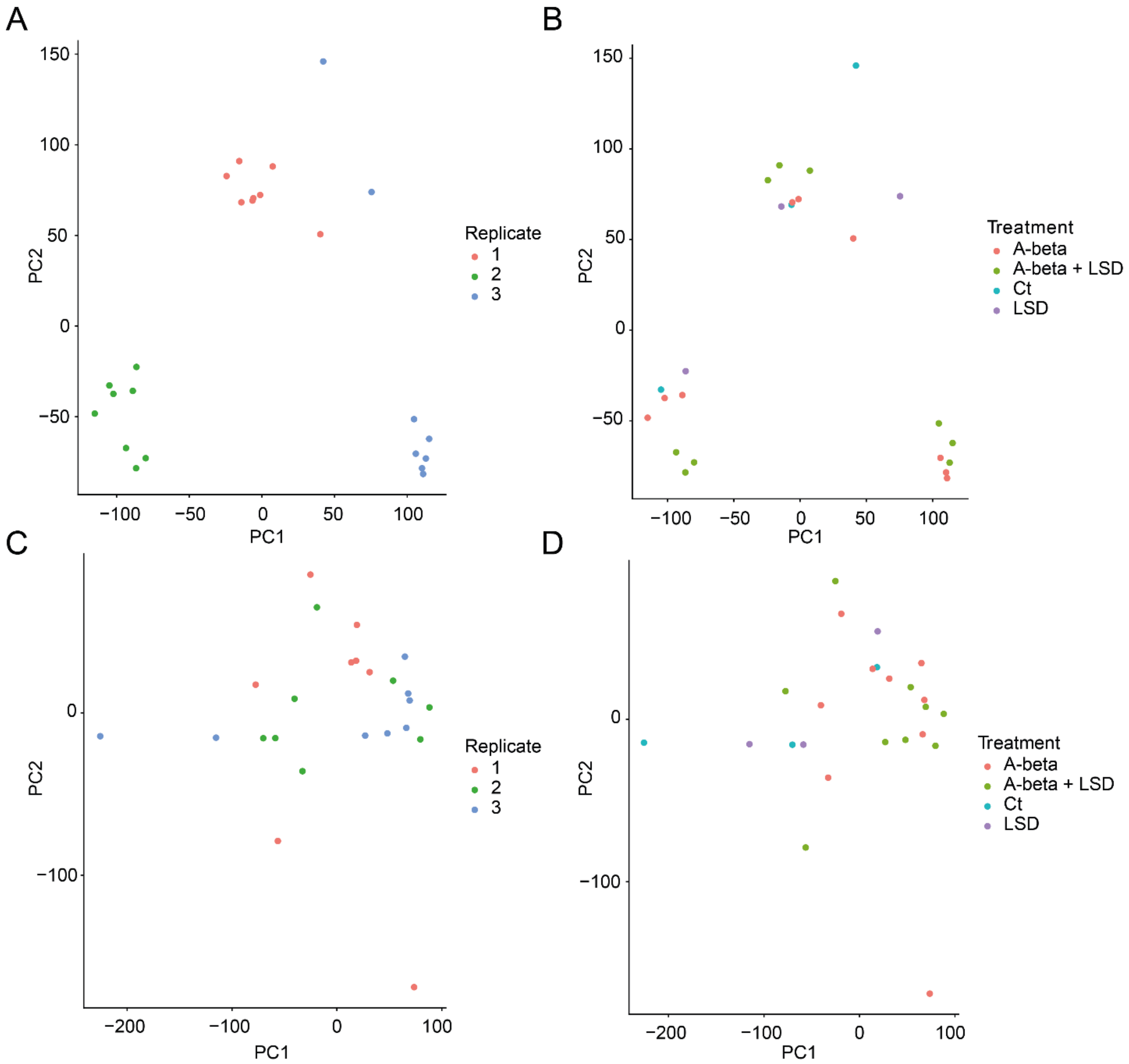

Figure S15. Principal component analysis of in vitro RNA-seq samples based on gene expression pre (A,B) and post (C,D) correction for batch. Each dot is a sample. (A,C) are coloured according to replicate, (B,D) are coloured according to treatment.

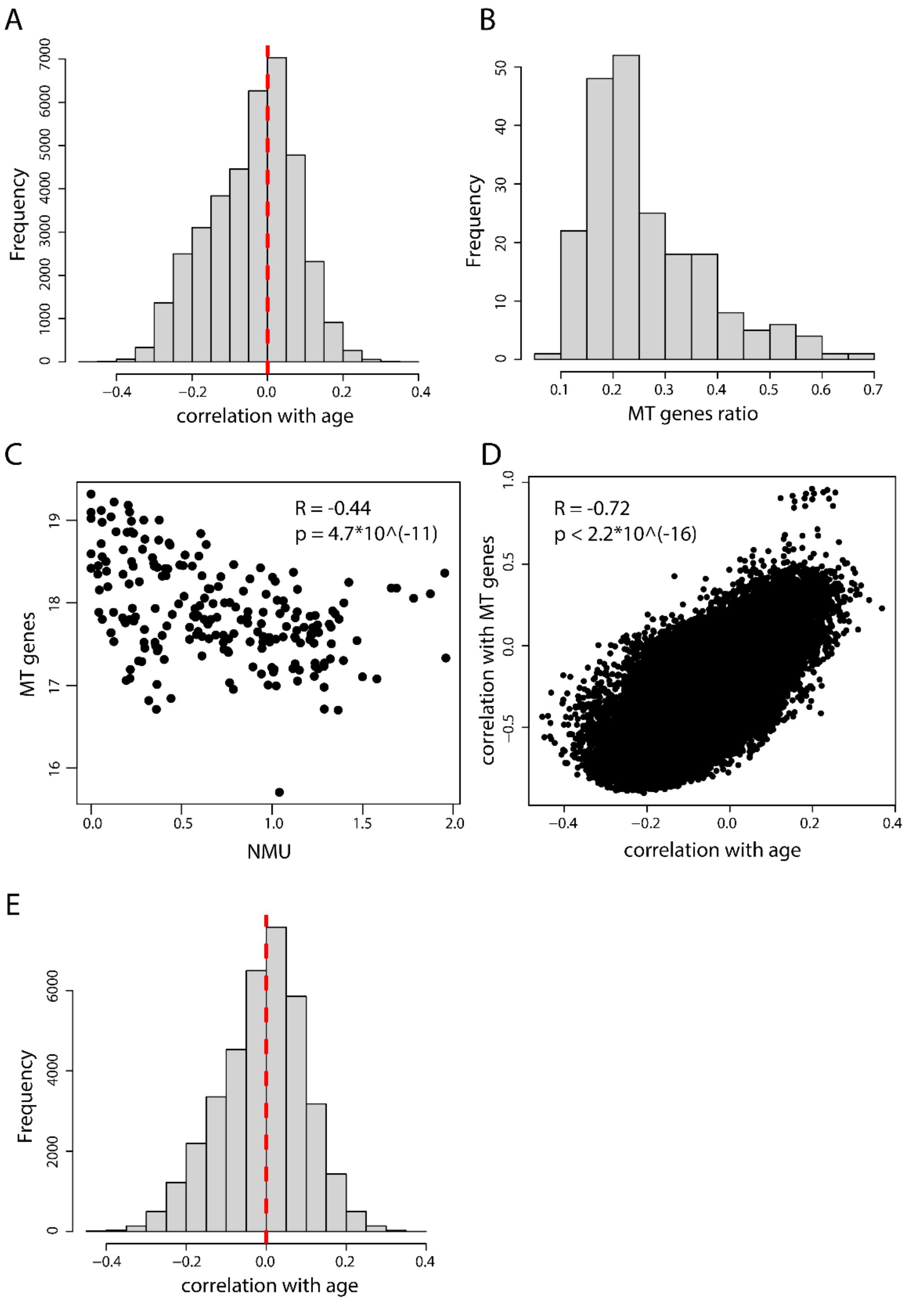

Figure S16. Correcting a normalization bias introduced by mitochondrial genes in the GTEx dataset. A. Distribution of gene expression - age correlations across GTEx frontal cortex samples; zero is indicated by a dashed red line. B. Fraction of reads mapping to mitochondrial genes over the total number of reads mapping to genes. C. Expression of NMU, the gene with the lowest correlation with age, vs mitochondrial genes across GTEx frontal cortex samples. D. Relationship between a gene's correlation with age and correlation with the expression of mitochondrial genes. E. Distribution of gene expression - age correlations across GTEx frontal cortex samples after removing the top 10% of samples with the highest expression of mitochondrial genes; zero is indicated by a dashed red line.

Supplementary Tables

no age	in vitro	no human samples	<10 samples	LCM	wrong tissue	neurodegenerative disease	repeated data	targeted screen	single cell
GSE120341	GSE102122	GSE99757	GSE15163	GSE12293	GSE127710	GSE129308	GSE113834	GSE11546	GSE178175
GSE120340	GSE40102	GSE29138	GSE159812	GSE12679	GSE146446	GSE135036	GSE106669	GSE118158	GSE81475
GSE182173	GSE63734	GSE4036	GSE26545		GSE165604	GSE135037	GSE161986	GSE27356	
GSE87610	GSE63738	GSE99757	GSE26586		GSE22569	GSE135751	GSE17757		
GSE53239	GSE163018		GSE100796			GSE136010	GSE22521		
GSE44771			GSE30352			GSE150696	GSE5392		
GSE44770			GSE44147			GSE159940			
GSE44768			GSE49379			GSE167490			
			GSE58604			GSE167494			
			GSE9335			GSE193391			
			GSE47966			GSE20168			
						GSE20295			
						GSE110731			
						GSE110732			
						GSE68719			
						GSE84422			

Table S1. Aging datasets retrieved by the search strategy described in the Supplementary Methods but excluded from the analysis. Gene Expression Omnibus identifiers are grouped according to the reason for exclusion, as indicated by the column headers. LCM = laser capture microdissection.

Dataset	Platform	Reference
**GSE101521**	RNA-seq	*Pantazatos et al. 2017*
**GSE102556**	RNA-seq	*Labonté et al. 2017*
**GSE102741**	RNA-seq	*Wright et al. 2017*
**GSE106670 (GSE106669)**	RNA-seq	*Cheng et al. 2018*
**GSE113842 (GSE113834)**	Affymetrix Human Gene Expression Array	*Parras et al. 2018*
**GSE11512**	Affymetrix GeneChip Human Genome U133 Plus 2.0 Array	*Somel et al. 2009*
**GSE125681**	Illumina HumanHT-12 V4.0 expression beadchip	*Cabrera-Mendoza et al. 2020*
**GSE13564**	Affymetrix Human Genome U133 Plus 2.0 Array	*Breen et al. 2018*
**GSE161986**	Affymetrix Human Genome U133A 2.0 Array	*Vornholt et al. 2020*
**GSE174409**	RNA-seq	*Seney et al. 2021*
**GSE17612**	Affymetrix Human Genome U133 Plus 2.0 Array	*Maycox et al. 2009*
**GSE21138**	Affymetrix Human Genome U133 Plus 2.0 Array	*Narayan et al. 2008*
**GSE21935**	Affymetrix Human Genome U133 Plus 2.0 Array	*Barnes et al. 2011*
**GSE22570**	Affymetrix Human Gene 1.0 ST Array [transcript (gene) version]	*Dönertaş et al. 2017*

**GSE37981**	Affymetrix Human X3P Array	*Pietersen et al. 2014*
**GSE49376**	Illumina HumanHT-12 V4.0 expression beadchip (gene symbol)	*Xu et al. 2014*
**GSE51264**	RNA-seq	*He et al. 2014*
**GSE5388**	Affymetrix Human Genome U133A Array	*Ryan et al. 2006*
**GSE5390**	Affymetrix Human Genome U133A Array	*Lockstone et al. 2007*
**GSE59288**	RNA-seq	*Liu et al. 2016*
**GSE59630**	Affymetrix Human Exon 1.0 ST Array [transcript (gene) version]	*Olmos-Serrano et al. 2016*
**GSE60190**	Illumina HumanHT-12 V3.0 expression beadchip	*Jaffe et al. 2014*
**GSE64810**	RNA-seq	*Labadorf et al. 2015*
**GSE71620**	Affymetrix Human Gene 1.1 ST Array [transcript (gene) version]	*French et al. 2017*
**GSE92538**	Affymetrix Human Genome U133A Array	*Hagenauer et al. 2018*
**GSE99349**	RNA-seq	*Ribeiro et al. 2017*
**GTEx**	RNA-seq	
**GSE33000**	Rosetta/Merck Human 44k 1.1 microarray	*Narayanan et al. 2014*
**GSE30272**	Illumina Human 49K Oligo array	*Colantuoni et al. 2011*

Table S2. Aging transcriptomic datasets included in the analysis.

Category	# of samples
**Gender**	
Male	2032
Female	1018
**Region**	
DLPFC	1335
OFC	480
PFC	783
SFG	84
TC	72
CBC	44
VFC	14
Other	274
**Diagnosis**	
Healthy	1863
MDD	237
BD	84
SCZ	80
DS	65
AUD	49
ASD	47
OUD	40
HD	177
CUD	18
OCD	9
ED	3
AD	310
**Ethnicity**	
Caucasian	918
African American	275
**Alcohol**	
Yes	86
No	173
**Smoke**	
Yes	213
No	310

Table S3. Number of samples of the ageing datasets considered in our analysis across annotated features.

Table S4. GSEA results for each combination of aging dataset, intervention dataset and direction of differential expression

Dataset	Drug	Model	Treatment regime	Sampling time	Reference
Not deposited	MDMA	M. musculus	11 consecutive days	8h after last administration	*Fernàndez-Castillo et al. 2012*
GSE161626	DOI	M. musculus	Single administration	24h, 48h, 7days	*de la Fuente Revenga et al. 2021*
GSE81672	Ketamine	M. musculus	Single administration		*Bagot et al. 2017*
GSE26364	Ketamine	M. musculus	Long term		
GSE209859	Psilocybin	M. musculus	Single administration	3 hours, 4 weeks	*Fadahunsi et al. 2022*
GSE23728 (GSE14720)	DOI	R. norvegicus	Single administration	2 hours	
GSE179378	LSD	R. norvegicus	Single administration	90 min	Nichols & Sanders-Bush 2002
GSE179379	LSD	R. norvegicus	Chronic treatment	3 weeks after withdrawal	*Martin et al. 2014*
Not deposited	DMT	R. norvegicus	Every other day for 5 days	72h after last administration	*Kelley et al. 2022*
Not deposited	Harmaline	R. norvegicus	Every other day for 5 days	72h after last administration	*Kelley et al. 2022*
Not deposited	Pharmahuasca	R. norvegicus	Every other day for 5 days	72h after last administration	*Kelley et al. 2022*

Table S5. Psychoplastogen datasets included in the analysis

Dataset	Comparator	Label
GSE64607	Aging-Reverting	Exercise (28d)
GSE164798	Aging-Reverting	Exercise (30d)
GSE111273	Aging-Reverting	EE Young / EE Old
GSE72507	Aging-Concordant	Alcohol (VC)
GSE60676	Aging-Concordant	Alcohol (VC 2)
GSE28515	Aging-Concordant	Alcohol (IP injection)
GSE75772	Aging-Concordant	rat aging

Table S6. Aging-Reverting and Aging-Concordant Comparator datasets included in the analysis, with corresponding labels used in Figure 1EF.

Table S7. Meta-analysed p-values and aging-opposition log p-value ratios (APR) for each intervention. APR values were capped at ±300 when aggregated p-values fell below floating-point precision. Infinite values for age-reversal p-value ratios were set to 300.

Table S8. Mean expression change of intervention-regulated genes used in the aging and dementia analyses.

Table S9. Cumulative transcriptional opposition scores of intervention-induced gene expression signatures relative to aging- and dementia-associated transcriptional changes.

Table S10. Intervention-regulated genes changing opposite to or concordantly with human brain aging signatures, per reference dataset.

Dataset	Disease	Reference
GSE110731	Alzheimer’s Disease	*Li et al. 2019*
GSE150696	Alzheimer’s Disease, dementia with Lewy bodies, Parkinson's disease dementia	*Low et al. 2021*
GSE33000	Alzheimer’s and Huntington’s Disease	*Narayanan et al. 2014*
GSE44770	Alzheimer’s Disease	*Zhang et al. 2013*
GSE53697	Alzheimer’s Disease	*Scheckel et al. 2016*
GSE84422	Alzheimer’s Disease	*Wang et al. 2016*

Table S11. Dementia datasets

Table S12. GSEA results for all combinations of dementia dataset, intervention dataset and direction of differential expression

Table S13. Meta-analyzed p-values and dementia-opposition p-value ratios for each intervention.

Table S14. Intervention-regulated genes changing opposite to or concordantly with dementia signatures, per reference dataset

Table S15. GSEA results for each combination of aging dataset, LSD concentration and direction of differential expression

Table S16. Meta-analyzed p-values and aging-opposition p-value ratios for each LSD concentration.

Table S17. Functional Enrichment for genes up-regulated by LSD and down-regulated upon aging

Table S18. Functional Enrichment for genes down-regulated by LSD and up-regulated upon aging

Table S19. Functional Enrichment for genes up-regulated by LSD and down-regulated in dementia

Table S20. Functional Enrichment for genes down-regulated by LSD and up-regulated in dementia

Table S21. Full list of Gene Ontology categories tested for differential expression in primary neurons treated with LSD plus Aβ relative to Aβ alone.

Supplementary Methods

Aging datasets collection and curation

We mined the Gene Expression Omnibus (GEO) database (Clough & Barrett 2016) for transcriptomic data of human prefrontal cortex (PFC) samples with available age annotation of the donors as follows. On the 7th of April 2022, we used the query (prefrontal cortex[All Fields] AND age[All Fields]) AND "Homo sapiens"[porgn] AND ("gse"[Filter] AND ("Expression profiling by array"[Filter] OR "Expression profiling by high throughput sequencing"[Filter])), retrieving 95 raw GEO datasets with at least 10 samples (**File S1**), which we downloaded using the GEOquery R package (Davis & Meltzer 2007). We processed Affymetrix arrays with the Robust Multichip Average (RMA) normalization (performing background correction and probe-level quantile normalization) and Illumina arrays with quantile normalization using negative and positive control probes (Stafford 2008). We mapped microarray probes to gene symbols using the respective platforms' information in GEO for each dataset, and when multiple probes matched the same gene symbol, we selected the probe with the highest mean expression across the considered dataset's samples. When data were not already log-transformed, we applied a $\log_2$ transformation, adding an offset of 1.

We thoroughly manually inspected each dataset and kept only data allowing computing correlation scores between sample age and individual genes' expression level across samples, removing samples for which age annotation was not reported, then filtering out datasets with less than 10 remaining samples per analysed brain region, or not including human samples or derived from in vitro models only (**Table S1**). We thus retained 28 datasets (**Table S2**) and included an external dataset from GTEx (GTEx Consortium 2013) comprising samples from 188 subjects transcriptionally profiled in the dorsolateral prefrontal cortex (DLPFC) (after quality filtering, see below).

We observed that several datasets included samples from very young subjects, thus being most useful for the study of brain development (**Figure S1**). Given our focus on aging, we kept datasets with at least 10 samples derived from adult subjects (>= 20 years). This left us with 24 datasets for a total of 3,086 samples spanning a wide range of ages (from 2 days to 106 years) and including also pre-natal samples. Despite our interest in the prefrontal cortex (PFC) of healthy adult subjects, we included in our collection all samples from the selected datasets, and extensively manually curated and harmonized the metadata, comprising annotation for gender, race, psychiatric diagnosis, and the dissected brain regions (**Table S3**,

Figure S1BC). Nevertheless, in our comparisons between aging and LSD transcriptional signatures, we only employed datasets annotated as DLPFC or PFC.

Quality filtering assessments led us to discard an outlier dataset showing a low similarity in gene-age relationships with the remaining 16 DLPFC and PFC datasets (GSE5388, **Figure S3A**) and a dataset with skewed correlation between gene expression and age (GSE102741, **Figure S3B**).

We downloaded GTEx data alongside sample age annotations from the GTEx Portal(GTEx Consortium 2013) on 20/09/2022 (files: 'gene_reads_2017-06-05_v8_brain_frontal_cortex_ba9.gct.gz' and 'https://storage.googleapis.com/gtex_analysis_v8/annotations/GTEx_Analysis_v8_Annotations_SubjectPhenotypesDS.txt' - currently available at https://storage.googleapis.com/adult-gtex/annotations/v8/metadata-files/GTEx_Analysis_v8_Annotations_SubjectPhenotypesDS.txt); we normalised RPM (reads per million), mapped gene identifiers to gene symbols as described above for other datasets, and log-transformed resulting data (with an offset of 1). We removed the 10% of samples with the highest expression of mitochondrial genes (identified based on the naming “MT-“). Before this correction, genes' correlation with age was skewed toward negative values (**Figure S16A**; 1st quartile:-0.12 , median: -0.03, 3rd quartile: 0.04). We observed that mitochondrial genes' reads represented a high proportion of reads in some samples, accounting for 65% of reads mapped to genes (**Figure S16B**) and we therefore reasoned that this could generate normalisation artefacts in these samples, potentially leading to spurious gene expression-age correlations. Indeed, genes that are negatively correlated with age are expressed at lower levels in samples with high levels of MT genes (**Figure S16CD**). Removing the top 10% of samples with the highest expression of MT genes, correlations' skewing was reduced (**Figure S16E**; 1st quartile: -0.08, median: 0.0008, 3rd quartile: 0.06). Finally, we retained genes with RPM>0 in at least 10 samples.

Comparing the aging-signature-opposition/-concordance of psychoplastogen with that of aging-reverting and aging-concordant comparators

We quantified the aging-signature opposition and concordance of each intervention by testing enrichment of treatment-induced gene sets along aging-associated ranked lists using a directional GSEA framework, followed by aggregation across datasets and comparison of opposition versus concordance signals.

For each intervention (psychoplastogens, enriched environment, exercise, ethanol, or rat aging signatures), we identified differentially expressed genes (DEGs; FDR < 0.05), separating up- and down-regulated genes. These gene sets were used independently as query signatures in GSEA against each aging-ranked list. GSEA yielded an enrichment score (ES) and an associated two-sided p-value for each query and dataset.

Under the aging-opposing hypothesis, treatment-induced up-regulated genes are expected to be enriched among genes whose expression decreases with age (i.e. at the bottom of the ranked list, corresponding to negative ES). In contrast, treatment-induced down-regulated genes are expected to be enriched among genes whose expression increases with age (i.e. at the top of the ranked list, corresponding to positive ES). Conversely, under an aging-concordant hypothesis, enrichment is expected in the opposite directions.

Because our hypotheses are directional, we converted the two-sided GSEA p-values into one-sided p-values using the two2one function from the metap R package. To incorporate directionality, we adjusted p-values based on the sign of the ES. Specifically, when the observed ES was inconsistent with the tested hypothesis (e.g. positive ES for up-regulated genes under the aging-opposing framework), the corresponding one-sided p-value was inverted ($1 - p$), thereby encoding direction into the statistical measure. For example, a two-sided p-value of 0.05 becomes 0.025 under directional consistency, but 0.975 if the ES sign contradicts the opposition hypothesis. This transformation ensures that small p-values consistently represent evidence in favor of the tested direction.

For each intervention and aging dataset, this procedure yielded two directional p-values (one for up-regulated genes and one for down-regulated genes). We then aggregated p-values across aging datasets using Fisher's method to obtain overall measures of evidence for aging-opposition, separately for up- and down-regulated genes.

To assess whether observed signals reflected true age-opposition rather than generic concordance with aging trajectories, we repeated the same procedure under the aging-concordant framework by inverting the directional expectations (i.e. switching the ES sign considered consistent with the hypothesis). This yielded aggregated aging-concordance p-values, again computed separately for up- and down-regulated gene sets.

Thus, for each intervention, we obtained four aggregated p-values: aging-signature opposition and aging-signature concordance statistics for up- and down-regulated gene sets.

Finally, to quantify the relative strength of opposition versus concordance effects, we computed the ratio between aggregated aging-signature opposition and aging-signature

concordance p-values. This ratio provides a directional reliability measure, where values favoring reversal indicate stronger support for opposition to aging-associated transcriptional programs.

Cumulative transcriptional opposition score

To complement the enrichment-based analyses with a measure of the magnitude of transcriptional opposition, we calculated a cumulative transcriptional opposition score for each intervention. The score was calculated separately for intervention-upregulated and intervention-downregulated genes and independently for each aging or dementia reference dataset.

For each differentially expressed gene, we first determined whether the intervention-induced change was opposite or concordant with the corresponding reference signature. In the aging analysis, the reference direction was defined by the sign of the correlation between gene expression and age within each human aging dataset. Thus, for example, a gene upregulated by an intervention and negatively correlated with age was classified as aging-opposing, whereas a gene upregulated by both the intervention and aging was classified as aging-concordant. In the dementia analysis, the reference direction was defined by the sign of the disease-versus-healthy gene-level logFC within each dementia dataset.

Genes classified as opposing the reference signature contributed positively to the score, whereas concordantly regulated genes contributed negatively. Each gene was weighted by the absolute magnitude of its intervention-induced logFC. The score was therefore calculated as:

$$\text{Score} = \Sigma_{g \in O} |\text{logFC}_g| - \Sigma_{g \in C} |\text{logFC}_g|$$

where *O* is the set of intervention-regulated genes changing in the direction opposite to the reference signature, *C* is the set of genes changing in the concordant direction, and *logFC* is the intervention-induced log fold change of each gene.

Positive scores therefore indicate that the cumulative magnitude of transcriptional changes opposing aging or dementia exceeds that of concordant changes, whereas negative scores indicate predominant concordance with the reference signature. A value of zero indicates equal cumulative opposing and concordant transcriptional change. Scores were calculated separately for intervention-upregulated and intervention-downregulated genes for each reference dataset and then averaged across the corresponding aging or dementia datasets for comparison across interventions.

Testing psychoplastogens transcriptional opposition to signatures of dementia

We searched the Gene Expression Omnibus for transcriptomic data of human prefrontal cortex (PFC) in dementia on the 1/4/2022. Our query was: (("dementia"[MeSH Terms] OR dementia[All Fields]) AND prefrontal cortex[All Fields]) AND "Homo sapiens"[porgn] AND ("gse"[Filter] AND ("Expression profiling by array"[Filter] OR "Expression profiling by high throughput sequencing"[Filter])).

The query yielded 20 datasets from which we retained bulk transcriptomic data of DLPFC finally considering 6 datasets (**Table S11**). On these, we tested the relationship between LSD-induced and dementia-related transcriptional changes.

For each dataset, we selected healthy and demented samples of the DLPFC or mPFC. We performed a single sample GSEA (using the GSVA R package (Hänzelmann et al. 2013)) for each selected sample and each considered list of genes, i.e. up-regulated and down-regulated upon treatment with psychoplastogens/ethanol/enriched environment/exercise (FDR<0.05), employing the datasets described in "Psychoplastogen's datasets" and "Selection of aging-reverting and aging-concordant comparators" sections of the methods. For each treatment, we then compared the single sample GSEA scores resulting from probing healthy and demented samples with a t-test and computed the Cohen's d to measure the effect size of the difference.

We then proceeded similarly as described in the "Testing aging-signature opposition potential " section of the Methods. Specifically, we merged the GSEA p-values using the Fisher method (Edwards 2005) (using the metap R package), thus computing global reliability scores for dementia-signature opposition and dementia-signature concordance effects of each intervention (still keeping up- and down-regulated genes upon treatment separated). Finally, we quantified the relative reliability of dementia-signature opposition for each treatment in comparison to the reliability of dementia-concordance of the same treatments. To this aim, we repeated the procedure testing the dementia concordance of each treatment, switching the Cohen's d sign. So, for each treatment, we obtained an overall dementia-signature opposition and dementia-signature concordance p-value based on up- or down-regulated genes. We then computed the ratio between the dementia-signature opposition and dementia-signature concordance p-values, obtaining the results shown in **Figure 1F**.

Functional enrichment (human datasets)

We tested the functional enrichment of genes correlated with aging, analysing one aging dataset at a time through a gene set enrichment analysis (GSEA) using as a background list the genes ranked based on the correlation between basal expression with age, and gene ontology (GO) Biological Processes as gene signature collection. Similarly, for dementia, within each dataset, we computed a gene-wise ranking statistic reflecting differential expression between the case and healthy groups. Specifically, for each gene we computed a t-statistic for the difference in means (case - healthy) using group-specific variances and sample sizes. For each ranked gene list, we performed GO Biological Process gene set enrichment. We merged the p-values of individual datasets (either for aging or for dementia) with the Fisher method and retained GO categories with enrichment FDR < 0.05, correcting p-values for multiple testing with the Benjamini-Hochberg correction (p.adjust function from the stats R package).

Functional enrichment (in vivo LSD treatment)

We considered genes differentially expressed upon LSD (at an FDR < 0.05) and performed an enrichment analysis for GO biological processes, separately for each LSD dataset (chronic treatment and acute treatment with 50, 100, 200, 500 ug/kg LSD). We retained only the GO categories significantly enriched in genes up-regulated across LSD datasets (and, conversely, enriched in genes down-regulated across LSD datasets), merging the p-values of individual datasets with the Fisher method and finally retaining GO categories with FDR < 0.05.

Finally, we combined the information on GO categories enriched for genes correlated with aging and for genes differentially expressed upon LSD and presented only the categories enriched for genes with an opposite trend upon aging and LSD (Figure 2). Similarly, we combined the GO categories enriched for genes changing in opposite directions with LSD and dementia, and showed them in **Figure S12**.

Functional enrichment (in vitro treatments)

To assess whether the Gene Ontology (GO) categories showing opposite-direction regulation between LSD and human dementia were also modulated by LSD in an in vitro

model of neurodegenerative stress, we performed gene set analysis on RNA-seq data from primary mouse cortical neurons treated with Aβ alone or with Aβ followed by LSD.

GO categories tested in this analysis were not selected a priori, but were derived directly from the cross-dataset LSD-dementia comparison, focusing on biological process terms that were significantly regulated in opposite directions by LSD in vivo and across human dementia transcriptomes ($FDR < 0.05$). For each selected GO term, the corresponding mouse orthologous genes were retrieved using the org.Mm.eg.db annotation. Only gene sets containing at least 10 expressed genes in the in vitro dataset were retained for downstream analysis.

Competitive gene set testing was performed using CAMERA (Correlation Adjusted MEan RAnk gene set test) as implemented in the limma package. CAMERA evaluates whether genes belonging to a predefined gene set are, on average, more differentially expressed than genes outside the set, while explicitly accounting for inter-gene correlation within each gene set, thereby reducing false positives associated with coordinated gene expression.

CAMERA was applied to log-transformed, ComBat-seq–adjusted gene expression values using a linear model contrasting Aβ + LSD versus Aβ treatment. Results are reported as direction of regulation, p-values, and Benjamini–Hochberg–adjusted false discovery rates (FDR). This analysis enabled us to directly test whether GO programs identified as showing opposite-direction regulation between LSD treatment in vivo and human dementia were concordantly modulated by LSD in neurons exposed to Aβ-induced stress.

List of Supplementary Items

Supplementary Figures

Figure S1. Samples and their annotations across the aging transcriptomic datasets.

Figure S2. Integration and batch correction of 11 transcriptomic DLPFC datasets.

Figure S3. Similarity and distribution of gene–age correlations across aging datasets and sample composition across annotated categories.

Figure S4. SVA correction reduces dataset-associated variation in the integrated DLPFC dataset.

Figure S5. Distribution of normalized enrichment scores across human aging datasets for each intervention.

Figure S6. Concordance of curated aging datasets with an independent published brain aging signature.

Figure S7. Distribution of aging-opposition GSEA p-values for Connectivity Map compounds based on drug-upregulated genes.

Figure S8. Distribution of aging-opposition GSEA p-values for Connectivity Map compounds based on drug-downregulated genes.

Figure S9. Cumulative transcriptional opposition (CTO) of intervention-induced gene expression signatures to aging- and dementia-associated transcriptional changes.

Figure S10. Distribution of Cohen's d values across dementia datasets for genes up-regulated or down-regulated by each intervention.

Figure S11. External validation of chronic LSD transcriptional opposition using independent aging datasets and the Mount Sinai Brain Bank cohort.

Figure S12. Top 20 Gene Ontology (GO) categories statistically enriched in the genes up-regulated by LSD and down-regulated in dementia, and in the genes down-regulated by LSD and up-regulated in dementia.

Figure S13. Number of significantly differentially expressed genes relative to control for Aβ alone and for the corresponding combined Aβ+LSD treatment across three Aβ concentrations (100 nM, 500 nM, 1 μM).

Figure S14. Gene set enrichment analysis of the Aβ-induced transcriptional signature across five human Alzheimer's disease brain datasets.

Figure S15. Principal component analysis of in vitro RNA-seq samples.

Figure S16. Correcting a normalization bias introduced by mitochondrial genes in the GTEx dataset.

Supplementary Tables

Table S1. Aging datasets retrieved by the search strategy described in the Supplementary Methods but excluded from the analysis.

Table S2. Aging transcriptomic datasets included in the analysis.

Table S3. Number of samples of the ageing datasets considered in our analysis across annotated features.

Table S4. GSEA results for each combination of aging dataset, intervention dataset and direction of differential expression.

Table S5. Psychoplastogen datasets included in the analysis.

Table S6. Aging-Reverting and Aging-Concordant Comparator datasets included in the analysis, with corresponding labels used in Figure 1EF.

Table S7. Meta-analysed p-values and aging-opposition log p-value ratios (APR) for each intervention.

Table S8. Mean expression change of intervention-regulated genes used in the aging and dementia analyses.

Table S9. Cumulative transcriptional opposition scores of intervention-induced gene expression signatures relative to aging- and dementia-associated transcriptional changes.

Table S10. Intervention-regulated genes changing opposite to or concordantly with human brain aging signatures, per reference dataset.

Table S11. Dementia datasets.

Table S12. GSEA results for all combinations of dementia dataset, intervention dataset and direction of differential expression.

Table S13. Meta-analyzed p-values and dementia-opposition p-value ratios for each intervention.

Table S14. Intervention-regulated genes changing opposite to or concordantly with dementia signatures, per reference dataset.

Table S15. GSEA results for each combination of aging dataset, LSD concentration and direction of differential expression.

Table S16. Meta-analyzed p-values and aging-opposition p-value ratios for each LSD concentration.

Table S17. Functional Enrichment for genes up-regulated by LSD and down-regulated upon aging.

Table S18. Functional Enrichment for genes down-regulated by LSD and up-regulated upon aging.

Table S19. Functional Enrichment for genes up-regulated by LSD and down-regulated in dementia.

Table S20. Functional Enrichment for genes down-regulated by LSD and up-regulated in dementia.

Table S21. Full list of Gene Ontology categories tested for differential expression in primary neurons treated with LSD plus Aβ relative to Aβ alone.

Supplementary Files

File S1. GEO search results for the 95 raw transcriptomic datasets retrieved by the aging-dataset search strategy.

File S2. R and package versions used for the analyses.

Supplementary Methods

Supplemental References

Bagot RC, Cates HM, Purushothaman I, Vialou V, Heller EA, Yieh L, LaBonté B, Peña CJ, Shen L, Wittenberg GM & Nestler EJ (2017) Ketamine and Imipramine Reverse Transcriptional Signatures of Susceptibility and Induce Resilience-Specific Gene Expression Profiles. *Biol. Psychiatry* 81, 285–295.

Barnes MR, Huxley-Jones J, Maycox PR, Lennon M, Thornber A, Kelly F, Bates S, Taylor A, Reid J, Jones N, Schroeder J, Scorer CA, Davies C, Hagan JJ, Kew JNC, Angelinetta C, Akbar T, Hirsch S, Mortimer AM, Barnes TRE & de Belleroche J (2011) Transcription and pathway analysis of the superior temporal cortex and anterior prefrontal cortex in schizophrenia. *J. Neurosci. Res.* 89, 1218–1227.

Breen MS, Ozcan S, Ramsey JM, Wang Z, Ma'ayan A, Rustogi N, Gottschalk MG, Webster MJ, Weickert CS, Buxbaum JD & Bahn S (2018) Temporal proteomic profiling of postnatal human cortical development. *Transl. Psychiatry* 8, 267.

Cabrera-Mendoza B, Fresno C, Monroy-Jaramillo N, Fries GR, Walss-Bass C, Glahn DC, Ostrosky-Wegman P, Genis-Mendoza AD, Martínez-Magaña JJ, Romero-Pimentel AL, Díaz-Otañez CE, García-Dolores F, González-Sáenz EE, Mendoza-Morales RC, Flores G, Vázquez-Roque R & Nicolini H (2020) Brain Gene Expression Profiling of Individuals With Dual Diagnosis Who Died by Suicide. *J. Dual Diagn.* 16, 177–190.

Cheng H, Xuan H, Green CD, Han Y, Sun N, Shen H, McDermott J, Bennett DA, Lan F & Han J-DJ (2018) Repression of human and mouse brain inflammaging transcriptome by broad gene-body histone hyperacetylation. *Proc. Natl. Acad. Sci. U. S. A.* 115, 7611–7616.

Clough E & Barrett T (2016) The Gene Expression Omnibus Database. *Methods Mol. Biol.* 1418, 93–110.

Colantuoni C, Lipska BK, Ye T, Hyde TM, Tao R, Leek JT, Colantuoni EA, Elkahloun AG, Herman MM, Weinberger DR & Kleinman JE (2011) Temporal dynamics and genetic control of transcription in the human prefrontal cortex. *Nature* 478, 519–523.

Davis S & Meltzer PS (2007) GEOquery: a bridge between the Gene Expression Omnibus (GEO) and BioConductor. *Bioinformatics* 23, 1846–1847.

Dönertaş HM, Fuentealba Valenzuela M, Partridge L & Thornton JM (2018) Gene expression-based drug repurposing to target aging. *Aging Cell* 17, e12819.

Dönertaş HM, İzgi H, Kamacıoğlu A, He Z, Khaitovich P & Somel M (2017) Gene expression reversal toward pre-adult levels in the aging human brain and age-related loss of cellular identity. *Sci. Rep.* 7, 5894.

Edwards AWF (2005) RA Fischer, statistical methods for research workers, (1925). In *Landmark writings in western mathematics 1640-1940*. Elsevier, pp.856–870.

Fadahunsi N, Lund J, Breum AW, Mathiesen CV, Larsen IB, Knudsen GM, Klein AB & Clemmensen C (2022) Acute and long-term effects of psilocybin on energy balance and feeding behavior in mice. *Transl. Psychiatry* 12, 330.

Fernàndez-Castillo N, Orejarena MJ, Ribasés M, Blanco E, Casas M, Robledo P, Maldonado R & Cormand B (2012) Active and passive MDMA (’ecstasy') intake induces differential transcriptional changes in the mouse brain. *Genes Brain Behav.* 11, 38–51.

French L, Ma T, Oh H, Tseng GC & Sibille E (2017) Age-Related Gene Expression in the Frontal Cortex Suggests Synaptic Function Changes in Specific Inhibitory Neuron Subtypes. *Front. Aging Neurosci.* 9, 162.

de la Fuente Revenga M, Zhu B, Guevara CA, Naler LB, Saunders JM, Zhou Z, Toneatti R, Sierra S, Wolstenholme JT, Beardsley PM, Huntley GW, Lu C & González-Maeso J (2021) Prolonged epigenomic and synaptic plasticity alterations following single exposure to a psychedelic in mice. *Cell Rep.* 37, 109836.

GTEx Consortium (2013) The Genotype-Tissue Expression (GTEx) project. *Nat. Genet.* 45, 580–585.

Hagenauer MH, Schulmann A, Li JZ, Vawter MP, Walsh DM, Thompson RC, Turner CA, Bunney WE, Myers RM, Barchas JD, Schatzberg AF, Watson SJ & Akil H (2018) Inference of cell type content from human brain transcriptomic datasets illuminates the effects of age, manner of death, dissection, and psychiatric diagnosis. *PLoS One* 13, e0200003.

Hänzelmann S, Castelo R & Guinney J (2013) GSVA: gene set variation analysis for microarray and RNA-seq data. *BMC Bioinformatics* 14, 7.

He Z, Bammann H, Han D, Xie G & Khaitovich P (2014) Conserved expression of lincRNA during human and macaque prefrontal cortex development and maturation. *RNA* 20, 1103–1111.

Jaffe AE, Deep-Soboslay A, Tao R, Hauptman DT, Kaye WH, Arango V, Weinberger DR, Hyde TM & Kleinman JE (2014) Genetic neuropathology of obsessive psychiatric syndromes. *Transl. Psychiatry* 4, e432.

Kelley DP, Venable K, Destouni A, Billac G, Ebenezer P, Stadler K, Nichols C, Barker S & Francis J (2022) Pharmahuasca and DMT Rescue ROS Production and Differentially Expressed Genes Observed after Predator and Psychosocial Stress: Relevance to Human PTSD. *ACS Chem. Neurosci.* 13, 257–274.

Labadorf A, Hoss AG, Lagomarsino V, Latourelle JC, Hadzi TC, Bregu J, MacDonald ME, Gusella JF, Chen J-F, Akbarian S, Weng Z & Myers RH (2015) RNA Sequence Analysis of Human Huntington Disease Brain Reveals an Extensive Increase in Inflammatory and Developmental Gene Expression. *PLoS One* 10, e0143563.

Labonté B, Engmann O, Purushothaman I, Menard C, Wang J, Tan C, Scarpa JR, Moy G, Loh Y-HE, Cahill M, Lorsch ZS, Hamilton PJ, Calipari ES, Hodes GE, Issler O, Kronman H, Pfau M, Obradovic ALJ, Dong Y, Neve RL, Russo S, Kazarskis A, Tamminga C, Mechawar N, Turecki G, Zhang B, Shen L & Nestler EJ (2017) Sex-specific transcriptional signatures in human depression. *Nat. Med.* 23, 1102–1111.

Li P, Marshall L, Oh G, Jakubowski JL, Groot D, He Y, Wang T, Petronis A & Labrie V (2019) Epigenetic dysregulation of enhancers in neurons is associated with Alzheimer’s disease pathology and cognitive symptoms. *Nat. Commun.* 10, 2246.

Liu X, Han D, Somel M, Jiang X, Hu H, Guijarro P, Zhang N, Mitchell A, Halene T, Ely JJ, Sherwood CC, Hof PR, Qiu Z, Pääbo S, Akbarian S & Khaitovich P (2016) Disruption of an

Evolutionarily Novel Synaptic Expression Pattern in Autism. *PLoS Biol.* 14, e1002558.

Lockstone HE, Harris LW, Swatton JE, Wayland MT, Holland AJ & Bahn S (2007) Gene expression profiling in the adult Down syndrome brain. *Genomics* 90, 647–660.

Low CYB, Lee JH, Lim FTW, Lee C, Ballard C, Francis PT, Lai MKP & Tan MGK (2021) Isoform-specific upregulation of FynT kinase expression is associated with tauopathy and glial activation in Alzheimer's disease and Lewy body dementias. *Brain Pathol.* 31, 253–266.

Martin DA, Marona-Lewicka D, Nichols DE & Nichols CD (2014) Chronic LSD alters gene expression profiles in the mPFC relevant to schizophrenia. *Neuropharmacology* 83, 1–8.

Maycox PR, Kelly F, Taylor A, Bates S, Reid J, Logendra R, Barnes MR, Larminie C, Jones N, Lennon M, Davies C, Hagan JJ, Scorer CA, Angelinetta C, Akbar MT, Hirsch S, Mortimer AM, Barnes TRE & de Belleroche J (2009) Analysis of gene expression in two large schizophrenia cohorts identifies multiple changes associated with nerve terminal function. *Mol. Psychiatry* 14, 1083–1094.

Narayanan M, Huynh JL, Wang K, Yang X, Yoo S, McElwee J, Zhang B, Zhang C, Lamb JR, Xie T, Suver C, Molony C, Melquist S, Johnson AD, Fan G, Stone DJ, Schadt EE, Casaccia P, Emilsson V & Zhu J (2014) Common dysregulation network in the human prefrontal cortex underlies two neurodegenerative diseases. *Mol. Syst. Biol.* 10, 743.

Narayan S, Tang B, Head SR, Gilmartin TJ, Sutcliffe JG, Dean B & Thomas EA (2008) Molecular profiles of schizophrenia in the CNS at different stages of illness. *Brain Res.* 1239, 235–248.

Nichols CD & Sanders-Bush E (2002) A single dose of lysergic acid diethylamide influences gene expression patterns within the mammalian brain. *Neuropsychopharmacology* 26, 634–642.

Olmos-Serrano JL, Kang HJ, Tyler WA, Silbereis JC, Cheng F, Zhu Y, Pletikos M, Jankovic-Rapan L, Cramer NP, Galdzicki Z, Goodliffe J, Peters A, Sethares C, Delalle I, Golden JA, Haydar TF & Sestan N (2016) Down Syndrome Developmental Brain Transcriptome Reveals Defective Oligodendrocyte Differentiation and Myelination. *Neuron* 89, 1208–1222.

Pantazatos SP, Huang Y-Y, Rosoklija GB, Dwork AJ, Arango V & Mann JJ (2017) Whole-transcriptome brain expression and exon-usage profiling in major depression and suicide: evidence for altered glial, endothelial and ATPase activity. *Mol. Psychiatry* 22, 760–773.

Parras A, Anta H, Santos-Galindo M, Swarup V, Elorza A, Nieto-González JL, Picó S, Hernández IH, Díaz-Hernández JI, Belloc E, Rodolosse A, Parikshak NN, Peñagarikano O, Fernández-Chacón R, Irimia M, Navarro P, Geschwind DH, Méndez R & Lucas JJ (2018) Autism-like phenotype and risk gene mRNA deadenylation by CPEB4 mis-splicing. *Nature* 560, 441–446.

Pietersen CY, Mauney SA, Kim SS, Lim MP, Rooney RJ, Goldstein JM, Petryshen TL, Seidman LJ, Shenton ME, McCarley RW, Sonntag K-C & Woo T-UW (2014) Molecular profiles of pyramidal neurons in the superior temporal cortex in schizophrenia. *J. Neurogenet.* 28, 53–69.

Ribeiro EA, Scarpa JR, Garamszegi SP, Kasarskis A, Mash DC & Nestler EJ (2017) Gene Network Dysregulation in Dorsolateral Prefrontal Cortex Neurons of Humans with Cocaine Use Disorder. *Sci. Rep.* 7, 5412.

Ryan MM, Lockstone HE, Huffaker SJ, Wayland MT, Webster MJ & Bahn S (2006) Gene expression analysis of bipolar disorder reveals downregulation of the ubiquitin cycle and

alterations in synaptic genes. *Mol. Psychiatry* 11, 965–978.

Scheckel C, Drapeau E, Frias MA, Park CY, Fak J, Zucker-Scharff I, Kou Y, Haroutunian V, Ma'ayan A, Buxbaum JD & Darnell RB (2016) Regulatory consequences of neuronal ELAV-like protein binding to coding and non-coding RNAs in human brain. *Elife* 5. Available at: http://dx.doi.org/10.7554/eLife.10421.

Seney ML, Kim S-M, Glausier JR, Hildebrand MA, Xue X, Zong W, Wang J, Shelton MA, Phan BN, Srinivasan C, Pfenning AR, Tseng GC, Lewis DA, Freyberg Z & Logan RW (2021) Transcriptional Alterations in Dorsolateral Prefrontal Cortex and Nucleus Accumbens Implicate Neuroinflammation and Synaptic Remodeling in Opioid Use Disorder. *Biol. Psychiatry* 90, 550–562.

Somel M, Franz H, Yan Z, Lorenc A, Guo S, Giger T, Kelso J, Nickel B, Dannemann M, Bahn S, Webster MJ, Weickert CS, Lachmann M, Pääbo S & Khaitovich P (2009) Transcriptional neoteny in the human brain. *Proc. Natl. Acad. Sci. U. S. A.* 106, 5743–5748.

Stafford P (2008) *Methods in Microarray Normalization*, CRC Press.

Vornholt E, Drake J, Mamdani M, McMichael G, Taylor ZN, Bacanu S-A, Miles MF & Vladimirov VI (2020) Network preservation reveals shared and unique biological processes associated with chronic alcohol abuse in NAc and PFC. *PLoS One* 15, e0243857.

Wang M, Roussos P, McKenzie A, Zhou X, Kajiwara Y, Brennand KJ, De Luca GC, Crary JF, Casaccia P, Buxbaum JD, Ehrlich M, Gandy S, Goate A, Katsel P, Schadt E, Haroutunian V & Zhang B (2016) Integrative network analysis of nineteen brain regions identifies molecular signatures and networks underlying selective regional vulnerability to Alzheimer's disease. *Genome Med.* 8, 104.

Wright C, Shin JH, Rajpurohit A, Deep-Soboslay A, Collado-Torres L, Brandon NJ, Hyde TM, Kleinman JE, Jaffe AE, Cross AJ & Weinberger DR (2017) Altered expression of histamine signaling genes in autism spectrum disorder. *Transl. Psychiatry* 7, e1126.

Xu H, Wang F, Liu Y, Yu Y, Gelernter J & Zhang H (2014) Sex-biased methylome and transcriptome in human prefrontal cortex. *Hum. Mol. Genet.* 23, 1260–1270.

Zhang B, Gaiteri C, Bodea L-G, Wang Z, McElwee J, Podtelezhnikov AA, Zhang C, Xie T, Tran L, Dobrin R, Fluder E, Clurman B, Melquist S, Narayanan M, Suver C, Shah H, Mahajan M, Gillis T, Mysore J, MacDonald ME, Lamb JR, Bennett DA, Molony C, Stone DJ, Gudnason V, Myers AJ, Schadt EE, Neumann H, Zhu J & Emilsson V (2013) Integrated systems approach identifies genetic nodes and networks in late-onset Alzheimer's disease. *Cell* 153, 707–720.
