# Supplementary material for: Lysergic Acid Diethylamide Induces Transcriptional Changes Opposing Signatures of Brain Ageing and Neurodegeneration
Source: Aging Cell. 2026 Nov 10;25(11):e70793. doi: 10.1111/acel.70793 (PMC13751324; doi:10.1111/acel.70793)
Supplement: Supplementary file 2 [file ACEL-25-e70793-s008.docx]

**Figure S1.** Samples and their annotations across the aging transcriptomic datasets. A. Distribution of sample age (years). B. Distribution of gender (M = Male, F = Female, NA = Not available). C. Distribution of sampled brain regions (DLPFC = dorsolateral prefrontal cortex; PFC = prefrontal cortex; OFC = orbitofrontal cortex; SFG = superior frontal gyrus; TC = temporal cortex; CBC = cerebellar cortex; VFC = ventral frontal cortex). D. Number of samples by age (columns) within each dataset (rows).

**Figure S2.** Integration and batch correction of 11 transcriptomic DLPFC datasets.
A-C. Principal component analysis before batch correction. A. Samples projected onto the first two principal components (PC1 and PC2), coloured by dataset of origin, showing a strong dataset-associated batch effect. B. The same PCA projection, with samples coloured according to subject age. C. Relationship between subject age and PC1 before correction, showing little association between the principal source of transcriptional variation and age.
D-F. Corresponding analyses after batch correction. D. Samples coloured by dataset of origin show substantially reduced dataset-specific clustering. E. PCA projection coloured according to subject age. F. Relationship between subject age and PC1 after correction, revealing a significant positive correlation (Pearson’s *R* = 0.60, *p* < 2.2 × 10^-16^).

**Figure S3.** A. Similarity (Pearson’s correlation) between genes’ correlation with age in different ageing datasets. B. Distribution of genes’ correlation with age in different ageing datasets (median in red). C. Number of samples in the ageing datasets collection belonging to each combination of categories. DLPFC = dorsolateral prefrontal cortex; mPFC = medial prefrontal cortex; OFC = orbitofrontal cortex; SFG = superior frontal gyrus; TC= temporal cortex, CBC = cerebellar cortex; VFC = ventral frontal cortex; AD = Alzheimer disease; MDD = major depressive disorder; HD = Huntington disease; BD = bipolar disorder; SCZ = schizophrenia; DS =Down syndrome; AUD = alcohol use disorder; ASD = autism spectrum disorder; OUD = opioid use disorder; CUD = cannabis use disorder; OCD = obsessive compulsive disorder; ED = eating disorder.

**Figure S4**. SVA correction reduces dataset-associated variation in the integrated DLPFC dataset. A. Distribution across 4,893 genes of the fraction of expression variance uniquely associated with the dataset of origin. B. Distribution across the same genes of the fraction of expression variance uniquely associated with age after accounting for dataset of origin. C. Residual dataset-specific structure assessed by K-nearest-neighbour classification of dataset of origin. Points represent mean balanced accuracy across folds for each of 20 repeated cross-validation runs; the dashed line indicates chance-level balanced accuracy (1/11).

**Figure S5.** Distribution of normalized enrichment scores across human aging datasets for each intervention. A, B. Distribution of normalized enrichment scores (NES) across human aging datasets for genes upregulated (A) or downregulated (B) by each intervention. Each point represents one aging dataset, and boxplots summarize the distribution across datasets. Interventions are ordered by mean NES within each panel. Colours denote intervention class: psychoplastogens in black, aging-reverting comparators in purple, and aging-concordant comparators in orange. C. Mean NES for intervention-upregulated genes plotted against mean NES for intervention-downregulated genes across aging datasets. Interventions in the upper-left quadrant display the strongest aging-opposing pattern, with upregulated genes enriched among genes that decrease with age (negative NES) and downregulated genes enriched among genes that increase with age (positive NES).

**Figure S6.** Concordance of curated aging datasets with an independent published brain aging signature. Gene Set Enrichment Analysis (GSEA) performed separately for each dorsolateral or medial prefrontal cortex aging dataset, with genes ranked according to the correlation between their expression and sample-age. Genes reported by Dönertaş et al. (Dönertaş et al. 2018) to increase or decrease in expression with age were used as separate query signatures. Red and blue curves indicate enrichment of age-upregulated and age-downregulated genes, respectively.

**Figure S7.** Distribution of aging-opposition GSEA p-values for Connectivity Map compounds based on drug-upregulated genes. For each human aging dataset, the black density curve shows the distribution of GSEA p-values obtained for compounds profiled in neural progenitor cells in the Connectivity Map, using genes upregulated by each compound as query signatures. P-values are shown as -log10(p). The dashed red line indicates the corresponding value obtained for the chronic LSD transcriptional signature.

**Figure S8.** Distribution of aging-opposition GSEA p-values for Connectivity Map compounds based on drug-downregulated genes. For each human aging dataset, the black density curve shows the distribution of GSEA p-values obtained for compounds profiled in neural progenitor cells in the Connectivity Map, using genes downregulated by each compound as query signatures. P-values are shown as -log10(p). The dashed red line indicates the corresponding value obtained for the chronic LSD transcriptional signature.

**Figure S9.** Cumulative transcriptional opposition (CTO) of intervention-induced gene expression signatures to aging- and dementia-associated transcriptional changes. A. CTO relative to human brain aging signatures. B. CTO relative to dementia-associated transcriptional signatures. For each intervention and reference dataset, genes were classified as opposing or concordant according to the direction of the intervention-induced logFC relative to the corresponding aging- or dementia-associated expression change. CTO was calculated separately for intervention-upregulated (x-axis) and intervention-downregulated (y-axis) genes. Red dashed lines indicate zero. EE = enriched environment.

**Figure S10.** A, B. Distribution of Cohen’s d values across dementia datasets for genes up-regulated (A) or down-regulated (B) by each intervention. Each point represents one dementia dataset/condition comparison, and boxplots summarize the distribution across datasets. Interventions are ordered by median Cohen’s d within each panel. Colors indicate the global signature class: psychoplastogens in black, aging-reverting interventions in purple, and aging-concordant comparators in orange. C. Mean Cohen’s d for up-regulated intervention genes versus mean Cohen’s d for down-regulated intervention genes across dementia datasets. Points represent interventions, colored according to the same signature classes as in panels (A,B) This summary highlights the relative positioning of psychoplastogens, aging-reverting comparators, and aging-concordant comparators with respect to dementia-associated transcriptional changes.

**Figure S11.** A. Gene set enrichment analysis of genes up- and down-regulated upon chronic LSD in rats (used as queries) along background ranked list of genes sorted based on the correlation between their expression and sampled subjects’ age in two additional datasets not included in the main analysis, used as external validations**.** B. Gene set enrichment analysis of genes up- and down- regulated upon chronic LSD in rats for genes with high correlation with age according to the Mount Sinai Brain Bank (left) and for genes with high correlation with the Braak score of Alzheimer severity in the same dataset (right).

**Figure S12.** Top 20 Gene Ontology (GO) categories statistically enriched in the genes up-regulated by LSD and down-regulated in dementia (A), and in the genes down-regulated by LSD and up-regulated in dementia (B). The colour in the heatmap indicates the GSEA p-value (-log10) with sign depending on the sign of the normalized enrichment score (NES). Red, highly significant positive enrichments;  blue, highly significant negative enrichments. The green bar on the left indicates the overall FDR (-log10) combining the p-values obtained for each dataset.

**Figure S13.** Number of significantly differentially expressed genes (DEGs; FDR < 0.05) relative to control for Aβ alone and for the corresponding combined Aβ+LSD treatment across three Aβ concentrations (100 nM, 500 nM, 1 µM). The p-value was computed using a paired Wilcoxon signed-rank test (one-sided), pairing each Aβ dose with its matched Aβ+LSD condition.

**Figure S14.** Gene set enrichment analysis (GSEA) was performed independently for five human brain datasets comparing Alzheimer’s disease (AD) versus healthy control samples. Genes were ranked by log2 fold change differential expression analysis (AD vs control). Enrichment curves show the running enrichment score for genes upregulated (red) or downregulated (blue) by Aβ treatment in vitro, after mapping to human orthologs. Positive enrichment indicates concordance between the in vitro Aβ signature and transcriptional changes observed in AD brain tissue, whereas negative enrichment indicates inverse regulation.

**Figure S15.** Principal component analysis of in vitro RNA-seq samples based on gene expression pre (A,B) and post (C,D) correction for batch. Each dot is a sample. (A,C) are coloured according to replicate, B,D) are coloured according to treatment.

**Figure S16.** Correcting a normalization bias introduced by mitochondrial genes in the GTEx dataset. A. Distribution of gene expression - age correlations across GTEx frontal cortex samples; zero is indicated by a dashed red line. B. fraction of reads mapping to mitochondrial genes over the total number of reads mapping to genes. C. Expression of NMU, the gene with the lowest correlation with age, vs mitochondrial genes across GTEx frontal cortex samples. D. Relationship between a gene’s correlation with age and correlation with the expression of mitochondrial genes. E. Distribution of gene expression - age correlations across GTEx frontal cortex samples after removing the top 10% of samples with the highest expression of mitochondrial genes; zero is indicated by a dashed red line.
